# miR-212/132-Enriched Extracellular Vesicles Promote Differentiation of Induced Pluripotent Stem Cells Into Pancreatic Beta Cells

**DOI:** 10.3389/fcell.2021.673231

**Published:** 2021-05-13

**Authors:** Chunyu Bai, Qiwei Ren, Haifeng Liu, Xiangchen Li, Weijun Guan, Yuhua Gao

**Affiliations:** ^1^Institute of Precision Medicine, Jining Medical University, Jining, China; ^2^Institute of Animal Sciences, Chinese Academy of Agricultural Sciences, Beijing China; ^3^College of Basic Medicine, Jining Medical University, Jining, China; ^4^Department of Laboratory Medicine, Affiliated Hospital of Jining Medical University, Jining, China; ^5^College of Animal Science and Technology, College of Veterinary Medicine, Zhejiang A&F University, Lin’an, China

**Keywords:** iPSCs, beta cells, differentiation, extracellular vesicles, miRNAs

## Abstract

Pancreatic beta cell transplantation is the ideal method for treatment of type 1 diabetes mellitus (T1DM), and the generation of beta cells from induced pluripotent stem cells (iPSCs) of patients is a promising strategy. In this study, we improved a previous strategy to produce beta cells using extracellular vesicles (EVs) derived from mature beta cells and differentiated beta cells from iPSCs (i-Beta cells), which secreted insulin under glucose stimulation *in vitro* and ameliorated hyperglycemia *in vivo*. Mechanistic analyses revealed that EV-carried microRNA (miR)-212/132 (EV-miR-212/132) directly bound to the 3′ UTR of FBW7 to prevent its translation and FBW7 combined with NGN3 to accelerate its proteasomal degradation. EV-miR-212/132 stabilized NGN3 expression to promote differentiation of endocrine cells from induced iPSCs. Moreover, NGN3 bound to PDX1 to enhance transcription of endogenous miR-212/132 and formed a positive regulatory circuit that maintained the functions of mature pancreatic beta cells.

**Conclusion:**

This study describes a novel approach for beta cell production and supports the use of iPSCs for cell replacement therapy of T1DM.

## Introduction

Pancreatic beta cells control glucose homeostasis, which have well-tuned machinery to sense glucose and secrete insulin. Type 1 diabetes mellitus (T1DM) results from autoimmune destruction of beta cells in pancreatic islets. Replacement of these pancreatic beta cells with healthy cells is the ideal therapy for T1DM. Therefore, production of pancreatic beta cells from stem cells is an attractive strategy. Induced pluripotent stem cells (iPSCs) are similar to embryonic stem cells with regard to self-renewal and multilineage differentiation potential *in vitro* and *in vivo*, which are reprogrammed from somatic cells by transfection of “Yamanaka factors” (Takahashi and Yamanaka, 2006). Therefore, iPSCs have provided a new prospect for patient-specific cell therapy and the development of regenerative medicine. A scalable differentiation protocol to generate glucose-responsive beta cells from iPSCs *in vitro* has been reported previously (Pagliuca et al., 2014), but this protocol is complicated to prepare the factors and small molecules used for differentiation of iPSCs.

Extracellular vesicles (EVs) are endosome-derived nanometer-scale vesicles (40–1000 nm in diameter) with a “dish” shape (Heijnen et al., 1999), which are secreted from many cell types and act as mediators of intercellular communication to recipient cells by delivering host cellular proteins, lipids, DNAs, and RNAs (Jiang et al., 2017). They transport bioactive cargos that can be transferred into recipient cells to influence cellular functions. Previously reported cell type-specific EVs trigger lineage-specific differentiation of stem cells. Huang et al. demonstrated that EVs derived from dental pulp cells cultured on under growth and odontogenic differentiation conditions induce odontogenic differentiation of naïve human dental pulp stem cells and human bone marrow-derived stromal cells (Huang et al., 2016). EVs secreted by beta cells contain the miRNA profile of the host cell, including let-7 (Krek et al., 2005; Lovis et al., 2008), miR-212 (Malm et al., 2016), miR-132 (Malm et al., 2016), miR-124a (Tang et al., 2009), miR-26 (Bai et al., 2017a), miR-24, miR-148 (Melkman-Zehavi et al., 2011), miR-204 (Roldo et al., 2006), miR-146a, miR-15a, miR-29a, miR-9, miR-16, and miR-34 (Rosero et al., 2010; Bai et al., 2017b), which are important regulators of the functional maintenance and differentiation of pancreatic beta cells. The characteristics of EVs prompted us to investigate whether EVs secreted by beta cells promote the pancreatic endocrine lineage differentiation of iPSCs. Meanwhile, we assumed that the EV-carried miRNAs play an important role in the differentiation of beta cells from iPSCs. The goal of our study was to gain further insight into the mechanisms underlying the differentiation of iPSCs into pancreatic beta cells and in particular to identify EV-carried miRNAs and their targets involved in the differentiation of beta cells.

In this study, we improved the previously described protocol (Pagliuca et al., 2014; Millman et al., 2016) for beta cell generation from iPSCs using EVs secreted by beta cells. We obtained beta cells that not only secreted insulin under glucose stimulation *in vitro* but also ameliorated hyperglycemia *in vivo*. Mechanistic analyses indicated that EVs carried miR-212/132 secreted by beta cells, which directly bound to the 3′ UTR of FBW7 to prevent its translation. FBW7 is a substrate recognition component of an evolutionarily conserved SCF-type ubiquitin ligase, which combined with NGN3 (an important transcription factor for beta cell differentiation from stem cells) to regulate its stability. This study describes a novel approach for beta cell production and supports the use of iPSCs for beta cell replacement therapy of type 1 diabetes mellitus.

## Results

### Generation of i-Beta Cells Using EV Induction

**FIGURE 1:**
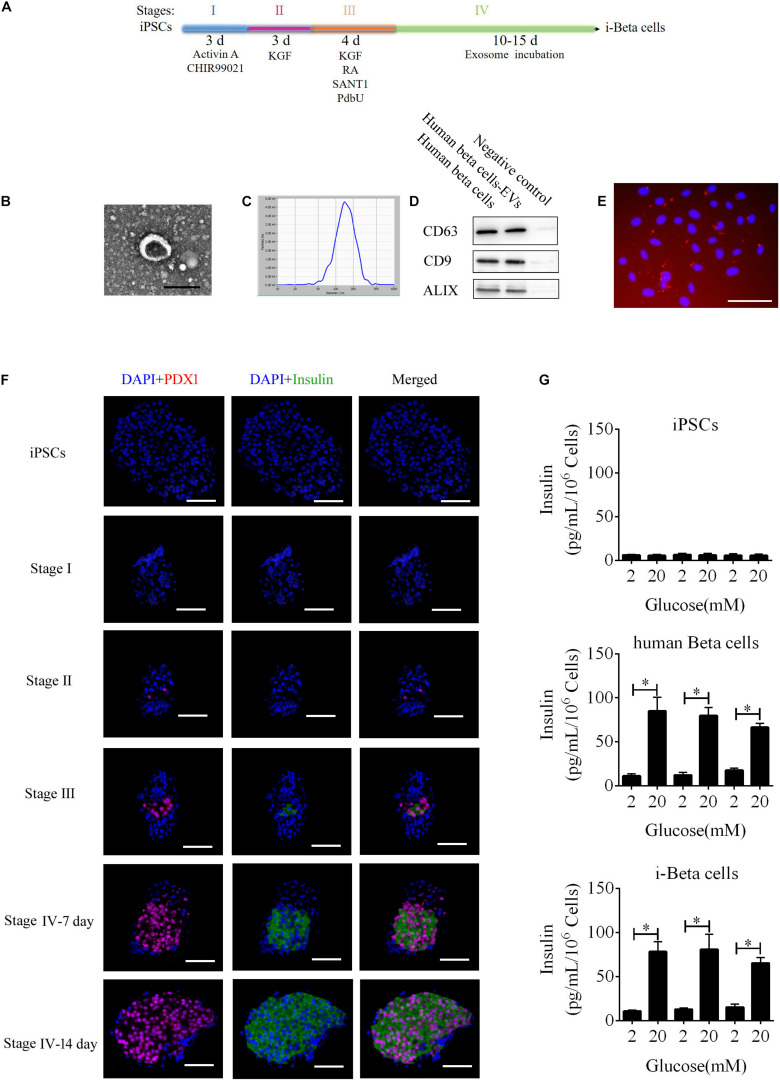
i-Beta cell differentiation from iPSCs using extracellular vesicles. **(A)** Schematic strategy for differentiation of iPSCs into i-Beta cells. EVs: extracellular vesicles derived from human beta cells. **(B)** Visualization of EVs from human beta cells by electron microscopy. Scale bar = 100 nm. **(C)** Size distributions of EVs from mature human beta cells. **(D)** Western blot analysis of EVs markers CD63, CD9, and ALIX. **(E)** Differentiated iPSCs were incubated with Dil-labeled EVs for 72 h and then red EV signals were detected by confocal microscopy. Scale bar = 100 μm. **(F)** Immunofluorescence microscopy was used to determine expression of PDX1 and Insulin during each stage of differentiation. Scale bar = 100 μm. **(G)** Glucose-stimulated insulin secretion of iPSCs, human beta cells, and i-Beta cells. An ELISA was used to measure secreted human insulin from cells stimulated sequentially with 2, 20, 2, 20, 2, and 20 mM glucose with 30 min of incubation at each concentration. Insulin was secreted from human beta cells and i-Beta cells but not iPSCs. *p < 0.05, **p < 0.001.

To improve a previous strategy for generation of beta cells from iPSCs, we added extracellular vesicles (EVs) derived from human beta cells to the protocol because of their biocharacteristics and developed a new strategy to differentiate iPSC into beta cells (i-Beta cells). The differentiation protocol was divided into four stages as shown in Figure 1A. First, the characteristics of EVs were analyzed by detecting specific proteins (CD63, CD9, and Alix) that indicated the presence of EV components using western blotting, observing the morphology of particles to clarify the integrity of EVs using transmission electron microscopy (TEM), and calculating the distribution of the diameter of particles and their number to show the characteristics of EV populations. EVs derived from human beta cells were collected; these exhibited good dispersion with a cup-shaped nanostructure with sizes ranging from 40 to 200 nm and contained characteristic EV marker proteins CD63, CD9, and Alix (Figures 1B,C). Moreover, the EVs were labeled with Dil and incubated with iPSCs. Dil was observed in differentiated iPSCs after 48 h (Figure 1E). These data revealed that the EVs efficiently delivered their biomolecules to iPSCs and assisted in differentiation into i-Beta cells. Next, we determined whether the biological characteristics of pancreatic beta cells were present in i-Beta cells. We observed the expression of pancreatic-duodenal homeobox factor 1 (PDX1) from stage II and insulin from stage III using immunofluorescence (Figure 1F). A critical functional feature of pancreatic beta cells is their ability to repeatedly perform glucose-stimulated insulin secretion (GSIS). The GSIS of i-Beta cells was detected by an ELISA, which demonstrated that these cells released insulin when treated with 2 and 20 mM glucose, whereas iPSCs did not release insulin *in vitro* (Figure 1G).

**FIGURE 2:**
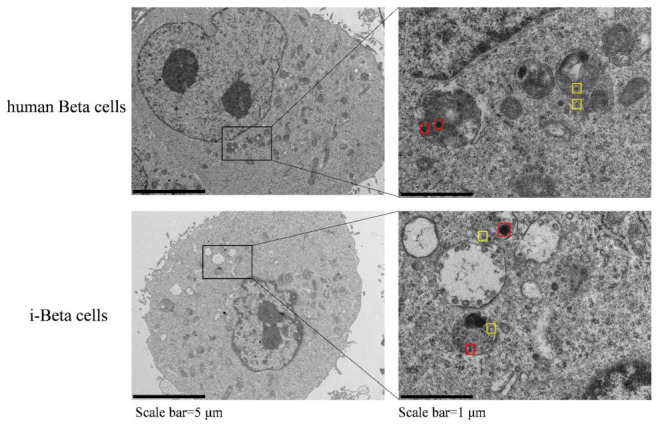
Electron microscopy of granules in sectioned cells with representative crystallized insulin granules (red) and early insulin granules (yellow) in human beta cells and i-Beta cells. Left panel shows electron microscopy images of granules in human beta cells (top image) and i-Beta cells (bottom image). Right panel shows a box and whisker plot of the number of insulin granules per cell (*n* = 20, Scale bar = 1 μm).

Insulin is packaged into secretory granules that initially appear as pale gray cores surrounded by a small electron-lucent area or light halo in pancreatic beta cells. The insulin condenses into granules with dark polygonal crystalline cores surrounded by a light halo (Deconinck et al., 1971; Like and Orci, 1972; Pagliuca et al., 2014). Next, we detected insulin granules by transmission electron microscopy and analyzed the amount of insulin granules per cell in human beta cells and i-Beta cells. Developing insulin granules and mature, crystallized insulin granules were observed in i-Beta cells and human beta cells with averages of 58 ± 9 and 68 ± 12 insulin granules per cell, respectively (Figure 2).

**FIGURE 3:**
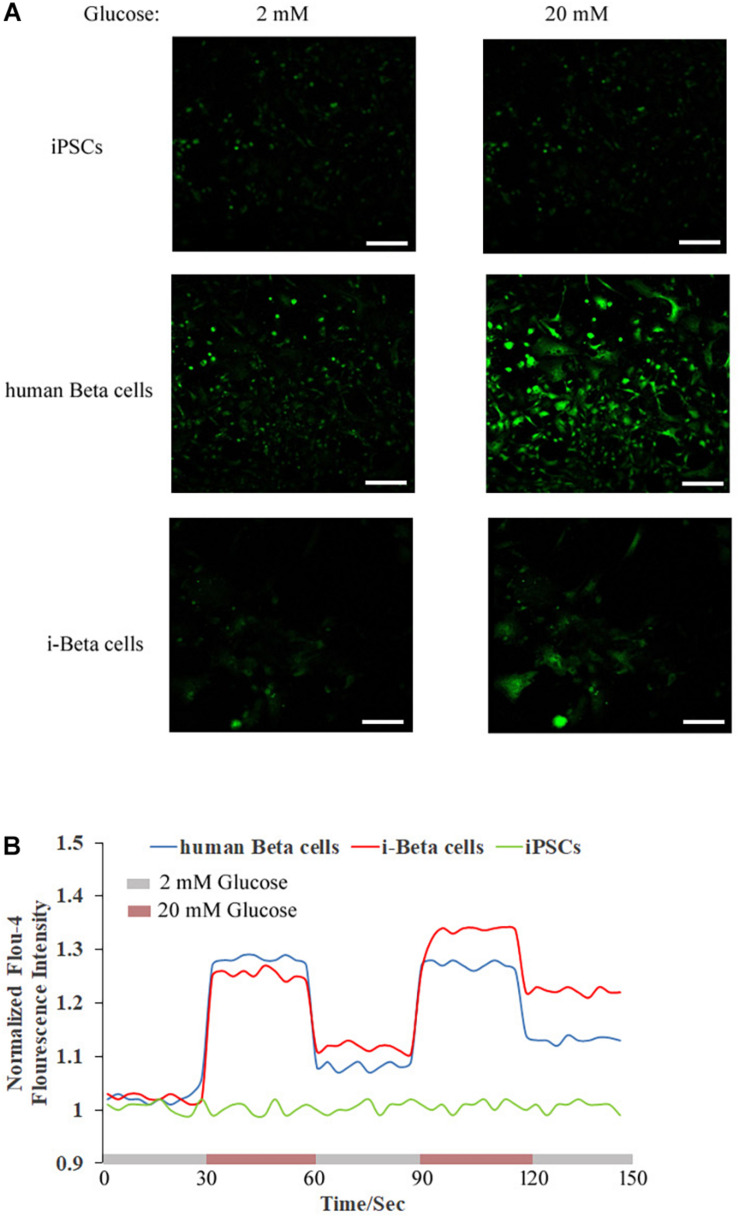
Cytosolic Ca^2+^ flux in response to multiple sequential high glucose stimulations. **(A)** Fluorescence images of Fluo-4 staining after treatment with various concentrations of glucose in human beta cells, i-Beta cells, and iPSCs. **(B)** Representative measurements of the dynamic normalized Fluo-4 fluorescence intensity in human beta cells, i-Beta cells, and iPSCs. i-Beta cells and human beta cells, but not iPSCs, responded to sequential glucose stimulations by increasing intracellular Ca^2+^.

**FIGURE 4:**
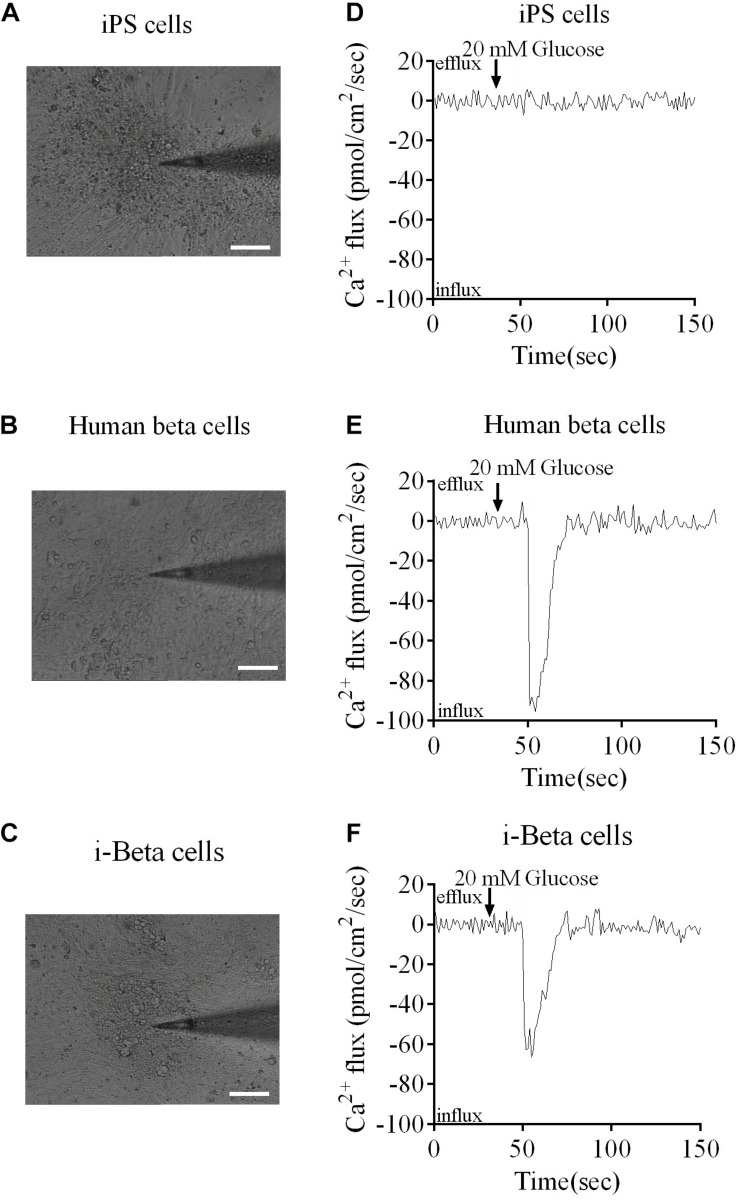
Ca^2+^ fluxes in human beta cells, i-Beta cells and iPSCs. **(A–C)** Images of human beta cells, i-Beta cells, and iPSCs analyzed by the non-invasive micro-test technique. **(D–F)**. Real-time normalized glucose-induced Ca^2+^ flux patterns of human beta cells, i-Beta cells, and iPSCs. Ca^2+^ influx was observed in human beta cells and i-Beta cells, but not iPSCs, after addition of 20 mM glucose.

Calcium plays an important role in releasing insulin from pancreatic beta cells, which sense changes in glucose levels through a calcium signaling pathway, and increasing glucose levels lead to membrane depolarization, causing an influx of calcium ions and triggering insulin exocytosis. Therefore, calcium flux was analyzed in i-Beta cells by Fluo-4 AM labeling and the non-invasive micro-test technique. We monitored calcium influx in i-Beta cells and iPSCs labeled with Fluo-4AM in real-time using a confocal optical system. The fluorescence value of Ca^2+^ showed that i-Beta cells and human beta cells responded to sequential glucose stimulations by increasing intracellular Ca^2+^ (Figure 3). Additionally, measurements of Ca^2+^ influxes were performed using the NMT technique. Prior to the detection, i-Beta cells were seeded in 35-mm dishes and perfused with a test solution containing 2 mM glucose and then a high concentration of glucose (20 mM). The results demonstrated that 20 mM glucose significantly increased the concentration of intracellular Ca^2+^ in i-Beta cells. Conversely, iPSCs displayed an abnormal calcium response (Figure 4). The bio-characteristic analysis of EV-induced i-Beta cells from iPSCs *in vitro* showed that these cells resemble stem-cell-derived beta cells (Zhang D. et al., 2009; Pagliuca et al., 2014) and human beta cells (Rosado-Olivieri et al., 2019).

### Functional Analysis of EV-Carried microRNAs in Differentiation of i-Beta Cells

**FIGURE 5:**
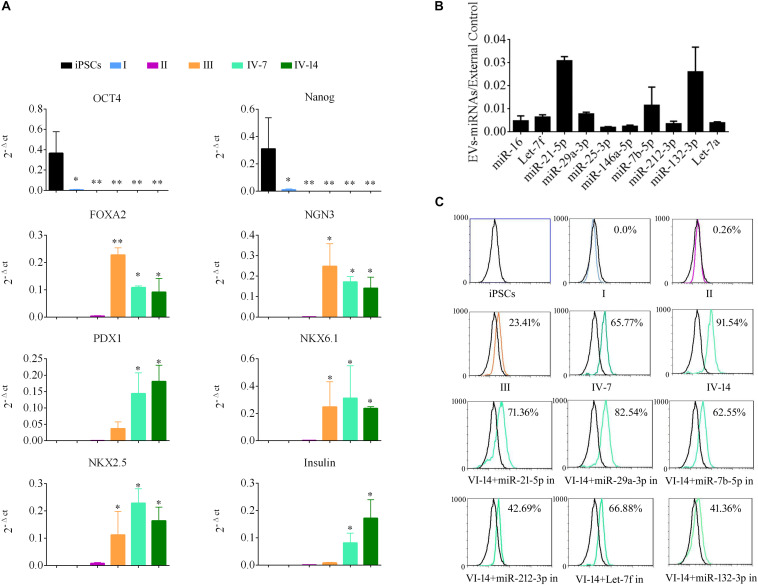
EV-carried microRNAs promote the differentiation of i-Beta cells from iPSCs. **(A)** Gene expression of iPSCs at various stages of differentiation compared with control iPSCs. Data are expressed as the mean ± SD of three independent experiments (*n* = 3). ^∗^*p* < 0.05, ^∗∗^*p* < 0.001 relative to control iPSCs. **(B)** The abundances of the top 10 miRNAs in EVs were verified by qPCR and expressed as the fold change versus the external control (*n* = 3). **(C)** Flow cytometric analysis of insulin expression during each stage of differentiation. Five miRNAs were selected from the top 10 miRNAs to analyze their roles in the formation of i-Beta cells from iPSCs. Insulin-positive cells were significantly decreased after addition of miR-212-3p and miR-132-3p inhibitors. in: miRNA inhibitor.

To demonstrate the role of EVs in differentiation of i-Beta cells, the mRNA levels of transcription factors were detected by qPCR at various stages, including Oct4 and Nanog, which are important to maintain the pluripotency of iPSCs, and FOXA2, PDX1, NKX6.1, NKX2.5, NGN3, and Insulin, which are important for development of pancreatic beta cells. Expression of pluripotency genes Oct4 and Nanog was decreased significantly and then disappeared at stage II. Moreover, FOXA2 expression was observed in stage II and continued to be upregulated in stage III and stage IV at 7 and 14 days, which is required for enhancer priming during pancreatic differentiation. During stage III and stage IV at 7 and 14 days, the differentiated cells rapidly began to express high levels of PDX1, NGN3, NKX6.1, and NKX2.5, while increasing expression of insulin (Figure 5A). The mRNA level of NGN3 was dramatically elevated from stage III and no significant changes were observed in stage IV at 7 and 14 days, but the protein level was increased significantly, which implied that post-translational modification regulated NGN3 stability.

MiRNAs participate in regulation of the differentiation and maturation of pancreatic beta cells and maintain their biological functions. To further analyze the functions of EVs, the miRNA profile was analyzed by RNA-seq. EV-carried miRNAs are listed in Supplementary Table 1. To verify the profile of miRNAs, the top 10 miRNAs, which were most enriched in EVs, were analyzed by qPCR (Figure 5B). miR-21-5p, miR-29a-3p, miR-7b-5p, Let-7f, and miR-212-3p/132-3p from the top 10 miRNAs were reported in previous studies to be involved in the development of pancreatic beta cells and the maintenance of their functions. Next, inhibitors of five miRNAs were applied to i-Beta cells combined with EVs. Insulin-positive cells were detected by flow cytometry, which indicated that the five miRNAs were involved in the formation of i-Beta cells from iPSCs, but the positive cells were significantly decreased after the addition of miR-212-3p and miR-132-3p inhibitors (Figure 5C and Supplementary Figure 1).

### EV-Carried miR-212/132 Regulate NGN3 Stability by Targeting FBW7

**FIGURE 6:**
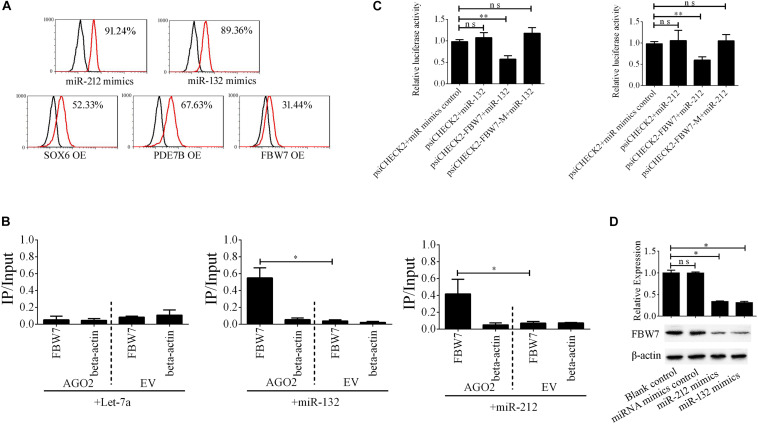
EV-miR-212/132 target FBW7 to increase formation of i-Beta cells from iPSCs. **(A)** Flow cytometric analysis of insulin-positive cells after overexpression of miR-212/132 or its targets in differentiated iPSCs in stage IV at day 7. The number of insulin-positive cells was significantly changed after overexpression of miR-212/132 mimics or FBW7, but not SOX6 or PDE7B, in stage IV at 7 day. OE, overexpression. **(B)** Immunoprecipitation of Myc-tagged AGO2 from iPSCs cotransfected with Myc-AGO2 and either miR-212/132 or Let-7a (negative control). The empty vector (EV) served as the Myc-AGO2-related negative control. FBW7 and β-actin mRNA levels were quantified by qPCR and relative immunoprecipitate (IP)/input (total RNA) values were plotted. The results showed that miR-212/132 facilitated AGO2 association with FBW7. **(C)** The effect of miR-212/132 on FBW7 expression was validated using luciferase reporter assays. HEK293T cells were cotransfected with miR-212/132 mimics and a luciferase reporter vector. The vector contained either the wildtype (WT) miR-212/132-binding 3′ UTR region of FBW7 or a mutated (MUT) 3′ UTR region. Mutating the miR-212/132 target site in the 3′ UTR of FBW7 abolished the inhibition of luciferase activity by miR-212/132. Relative luciferase activity and qPCR values represent the mean ± SD of at least three replicates. **(D)** Western blot analysis of FBW7 in iPSCs following overexpression of miR-212/132. Overexpression of miR-212/132 inhibited endogenous expression of FBW7. β-Actin was used as an endogenous control. Values represent the mean ± SD of at least three replicates. *p < 0.05, **p < 0.001.

The miR-212/132 cluster includes miR-212 and miR-132 that are a clustered within 500 base pairs of each other at chromosome 17 in humans. To examine the role of EV-carried miR-212/132 in the formation of i-Beta cells, miR-212/132 mimics were applied to differentiated iPSCs and then the expression rate of insulin was analyzed by flow cytometry (Figure 6A). Differentiated iPSCs showed significant upregulation of insulin, which was reversed in the presence of miR-212 and miR-132 inhibitors. To further investigate the function of EV-miR-212/132 in the formation of i-Beta cells, their potential targets were predicted using TargetScan, miRDB, and StarBase as listed in Supplementary Table 2 and then SOX6 (Iguchi et al., 2005; Bai et al., 2017a), FBW7 (Sancho et al., 2014), and PDE7B (Dayeh et al., 2014), which participate in pancreatic development or influence pancreatic islet dysfunction, were selected for overexpression in stage IV to assess their function in the formation of i-Beta cells. FBW7, but not SOX6 or PDE7B, significantly reduced the number of insulin-positive cells, in stage IV at 7 days (Figure 6A and Supplementary Figure 1). To determine whether the predicted site in the 3′UTR of FBW7 was responsible for silencing gene expression by miR-212/132, AGO2-IP-qPCR and luciferase reporter assays were used for further analyses. We performed AGO2 immunoprecipitation in HEK293T cells containing either an AGO expression vector or empty vector and transiently coexpressing miR-212/132 or Let-7a (negative control). The binding site of Let-7a was not found in the 3′UTR of FBW7 by bioinformatics algorithms. Therefore, Let-7a was used as a negative control. FBW7 levels were analyzed by qPCR of the immunoprecipitated products. FBW7 was specifically enriched by more than six-fold in the presence of AGO2 in miR-212/132-transfected cells compared with the negative control (Let-7a, Figure 6B). The wildtype (WT) and mutated (MUT) FBW7 3′UTR region were cloned and ligated downstream of a luciferase reporter gene and then cotransfected into HEK293T cells with pre-miR-212, pre-miR-132, or a control precursor. In HEK293T cells transfected with pre-miR-212 or pre-miR-132 and pRL-FBW7-WT, luciferase activity was significantly decreased relative to that in cells cotransfected with the control precursor or mutated FBW7 3′UTR region (Figure 6C). Western blotting also revealed that FBW7 underwent dramatic downregulation after transfection of miR-212/132 mimics into iPSCs (Figure 6D). These results demonstrated that EV-miR-212/132 directly suppressed translation of FBW7 by targeting seed sequences in the 3′UTR.

**FIGURE 7:**
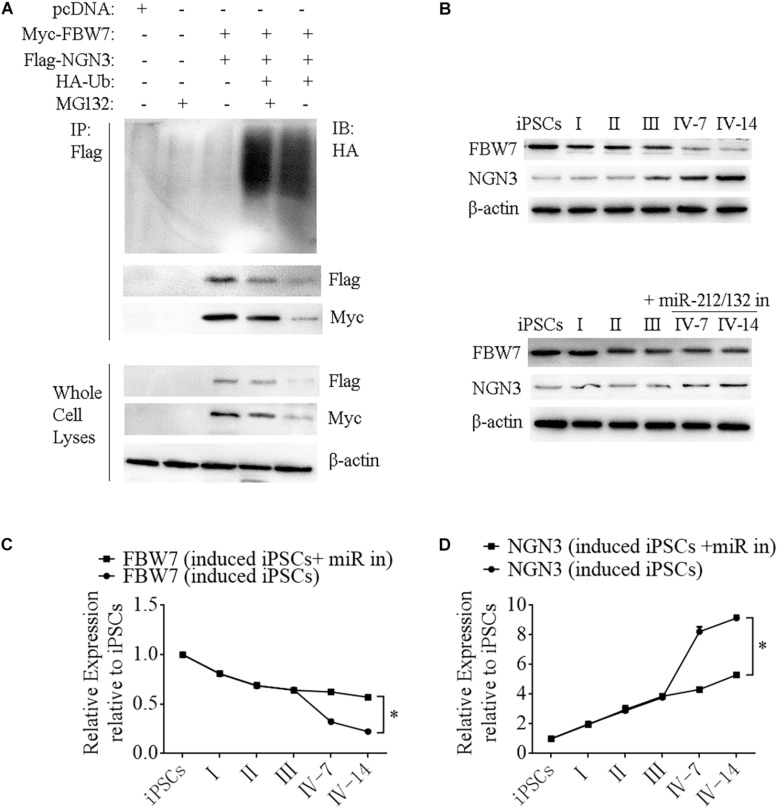
FBW7 combines with NGN3 to promote NGN3 degradation through ubiquitylation. **(A)** Immunoprecipitation-western blotting showed that FBW7 interacted with NGN3 and promoted NGN3 degradation through ubiquitylation. Polyubiquitylated NGN3 was dramatically elevated in MG132-treated cells transfected with FBW7. **(B–D)** Western blot analysis of FBW7 and NGN3 protein levels during formation of i-Beta cells after silencing miR-212/132. Graph shows mean FBW7 and NGN3 protein levels normalized to β-actin as the percentage of protein levels in iPSCs. Values represent the mean ± SD of at least three replicates.

Neurogenin 3 (Ngn3) is a basic helix-loop-helix protein transcription factor that binds to the E-box and is involved in pancreas development (Schwitzgebel et al., 2000; Grapin-Botton et al., 2001; Kaneto et al., 2005). Overexpressing Ngn3 in transgenic mice results in a marked increase in the formation of endocrine cells, which indicates that Ngn3 induces differentiation of islet cell precursors. FBW7 is a ubiquitin ligase that combines with NGN3 to form a degradation complex that influences NGN3 stability in mice (Sancho et al., 2014), but such regulation in human cells has not been fully investigated. We next constructed Myc-FBW7, Flag-NGN3, and HA-Ub plasmids and co-transfected them into HEK293T cells treated with 0 or 10 μM MG132 (proteasome inhibitor) to investigate the interaction between FBW7 with NGN3 and NGN3 degradation through ubiquitylation by immunoprecipitation (IP). MG132 suppressed FBW7-induced NGN3 degradation and ectopic FBW7 expression increased polyubiquitylated NGN3, which indicated that FBW7 promoted NGN3 polyubiquitylation (Figure 7A). To further analyze NGN3 degraded by FBW7, we investigated the dynamics of FBW7 and NGN3 at protein levels during the formation of i-Beta cells from iPSCs. IP data confirmed that NGN3 was increased with the induction time and FBW7 was decreased, but the opposite trend was found with ectopically expressed miR-212/132 inhibitors (Figures 7B–D). Combining our data with those from previous reports (Sancho et al., 2014), we found that Ngn3 stabilization after EV-miR-212/132-mediated Fbw7 loss contributed to a differentiation program, which induced iPSCs to differentiate into i-Beta cells.

### NGN3 Combines With PDX1 to Enhance Transcription of miR-212/132 by Forming a Regulatory Circuit

**FIGURE 8:**
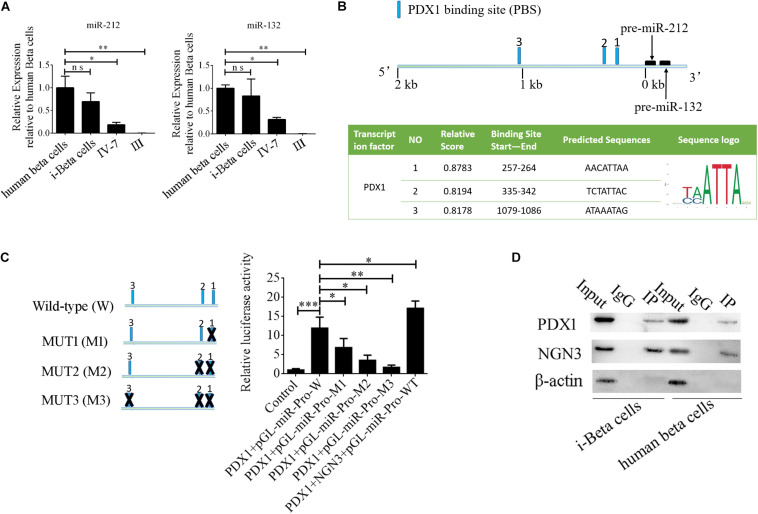
NGN3 interacts with PDX1 to enhance transcription of endogenous miR-212/132. **(A)** MiR-212/132 levels were measured by qPCR in human beta cells, i-Beta cells, and differentiated iPSCs in stage IV at day 7 and differentiated iPSCs in stage III. **(B)** Schematic of the predicted binding sites of PDX1 in the promoter region of miR-212/132 (top image). The promoter-binding sites for PDX1 were predicted by bioinformatics algorithms (bottom image). **(C)** The full-length miR-212/132 promoter (WT) and miR-212/132 promoter containing various mutated variants of PDX1-binding sites (MUT) were used to determine the effect on miR-212/132 expression. For these assays, WT and MUT vectors were cotransfected with PDX1 and PDX1/NGN3 into HEK293T cells. Results are expressed as relative luciferase activity and represent the mean ± SD of at least three replicates. **(D)** Immunoprecipitation-western blotting showed that NGN3 interacted with PDX1 in human beta cells and i-Beta cells.

MiR-212/132 play an important role in secretion of insulin from mature beta cells and miR-212/132 were maintained at higher levels after the formation of i-Beta cells (Figure 8A). Therefore, we assumed that endogenous miR-212/132 must be active. To determine the mechanisms underlying the activated transcription of miR-212/132 in differentiated iPSCs, we used JASPAR tools to screen for binding sites of the PDX1 transcription factor within the 2 kbp upstream region of pre-miR-212/132. Multiple binding sites were predicted, which could be bound by PDX1 (Figure 8B). Next, pGL3.0-miR-212/132 promotor vectors were constructed and mutations were introduced into PDX1-binding sites. The wildtype (WT) promotors of miR-212/132 or its mutated (MUT) counterparts together with PDX1 vectors were cotransfected into HEK293T cells. Then, luciferase activity was measured as a proxy for miR-212/132 expression. Our data showed that PDX1 bound to sequences within the miR-212/132 promoters to enhance their transcription (Figure 8C). PDX1 is essential for pancreas development and NGN3 combines with PDX1 directly or indirectly to enhance transcription of PDX1. We next assessed interactions between NGN3 with PDX1 by immunoprecipitation using an anti-PDX1 antibody in i-Beta cells and human beta cells. The results revealed that the PDX1/NGN3 complex was formed in these cells (Figure 8D). To assess whether NGN3 enhanced PDX1 transcription, we used the luciferase activity of pGL3.0-miR-212/132 promotor vectors. Luciferase activity was dramatically elevated after co-transfection of NGN3 with PDX1 (Figure 8C). Our data indicated that EV-carried miR-212/132 stabilized NGN3 expression to combine with PDX1 and enhance the transcription of miR-212/132 by forming a regulatory circuit.

### Cell Transplantation

**FIGURE 9:**
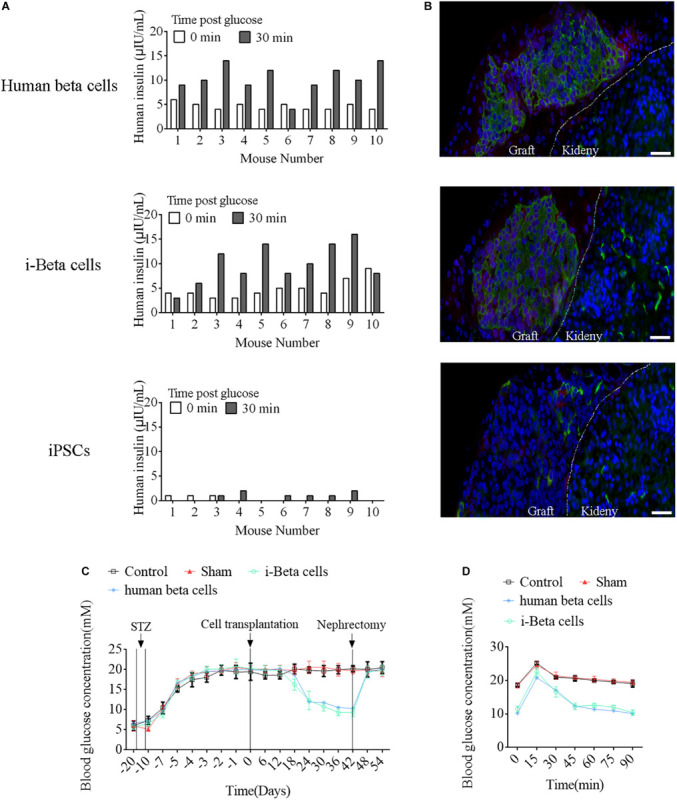
Functional analysis of i-Beta cells *in vivo* after transplantation. **(A)** ELISA detection of human insulin in serum of immunodeficient SCID mice transplanted with human beta cells, i-Beta cells, or iPSCs. Measurements were performed before (white bars) and at 30 min after (black bars) a glucose injection at 2 weeks post-transplantation. **(B)** Representative immunofluorescence images of kidneys at 2 weeks post-transplantation stained for insulin to confirm the presence of engrafted cells. Scale bar = 50 μm. **(C)** Transplanted human beta cells and i-Beta cells ameliorated hyperglycemia in diabetic mice. Mice were treated with mouse-specific beta cell toxin STZ (intraperitoneal injection for 6 days) to ablate endogenous beta cells. Human beta cells or i-Beta cells (2 × 10^6^ cells/mouse) were transplanted under the kidney capsule of diabetic mice. Fasting blood glucose was assessed every 6 days and a survival nephrectomy was performed on all mice on day 42 to remove the engrafted kidney. Unilateral nephrectomy of human beta cells or i-Beta cell graft-bearing mice resulted in a rapid rise in fasting blood glucose levels (*n* = 5). **(D)** Blood glucose levels were measured by a glucose tolerance test at 6 weeks post-transplantation. Human beta cells and i-Beta cells rapidly ameliorated hyperglycemia in diabetic mice (*n* = 5).

To assess the *in vivo* functions of i-Beta cells, 2 × 10^7^ i-Beta cells, human beta cells, or iPSCs were transplanted under the left kidney capsule of SCID mice. After a brief surgical recovery period (approximately 2 weeks), the mice were injected with (+)-D-glucose to induce hyperglycemia and serum was collected after 30 min to measure human insulin in serum using an ELISA. Insulin was secreted into the host bloodstream after transplantation of i-Beta cells and human beta cells. Conversely, no insulin was detected after transplantation of iPSCs (Figure 9A). At 4 weeks post-transplantation, the animals were sacrificed and engrafted kidneys were analyzed for insulin and GCG by immunofluorescence. The results showed the presence of pancreatic islet-like structures adjacent to the mouse kidney after transplantation of i-Beta cells and human beta cells (Figure 9B) but not iPSCs.

Next, we determined whether i-Beta cells functioned to control diabetic hyperglycemia. We transplanted 2 × 10^7^ i-Beta cells or human beta cells under the left kidney capsule of STZ-induced diabetic mice to analyze the utility of i-Beta cells to treat diabetes. As shown in Figure 9C, diabetic mice exhibited significantly reduced blood glucose levels at all time points after transplantation of i-Beta cells or human beta cells relative to control and sham groups. Additionally, after removal of kidneys transplanted with i-Beta cells or human beta cells, the blood glucose level of STZ-induced mice reverted to hyperglycemia within 3 days. Next, we assessed glucose clearance in the blood of diabetic mice after transplantation of i-Beta cells or human beta cells. Mice that received i-Beta cells also rapidly cleared glucose from their blood after a glucose injection at 30 days post-transplantation similarly to the glucose clearance found in diabetic mice transplanted with human beta cells (Figure 9D). Therefore, i-Beta cells, similar to normal human beta cells or other stem-cell-derived Beta cells (Shi et al., 2005; Enderami et al., 2017; Enderami et al., 2018b), were capable of secreting insulin and rapidly ameliorating hyperglycemia in a diabetic mouse.

## Discussion

Pancreatic beta cell transplantation to replace damaged cells is the ideal therapy for T1DM, but the shortage of donor cells restricts its application. Stem cells are an ideal cell source for transplantation, including embryonic stem cells (ESCs), iPSCs, and mesenchymal stem cells (MSCs). There is no apparent immune rejection of MSCs or iPSCs from patients after reintroduction. MSCs exist in bone marrow, adipose tissue, or other organs in adults, and are attractive donor cells for beta cell transplantation because they are multipotent and exert a strong immunoregulatory effect. However, the individualized MSCs obtained from T1DM patients have restricted application in cell replacement therapy. iPSCs are reprogrammed from skin fibroblasts or monocytes (Takahashi and Yamanaka, 2006; Jiang et al., 2014), cells that are relatively easy to obtain compared with MSCs, and are similar to embryonic stem cells with regard to self-renewal and multilineage differentiation potential *in vitro* and *in vivo*. T1DM patient-derived iPSCs, differentiated from pancreatic beta cells, are becoming important because of their potential for cell replacement therapy and drug screening, as well as improving our understanding of the pathophysiology of T1DM (Millman et al., 2016).

Many reports on the differentiation of insulin-producing cells and beta-like cells from iPSCs have indicated that miR-375 plays an important role in the development and insulin secretion of pancreatic beta cells (Poy et al., 2009; Enderami et al., 2017). Thus, miR-375 has been overexpressed in iPSCs or adult stem cells to induce the formation of insulin-producing cells (Lahmy et al., 2014; Piran et al., 2017), but the low efficiency of differentiation has limited the application of this approach. Previous reports provided a new approach to generate functional beta cells from iPSCs (Pagliuca et al., 2014; Millman et al., 2016; Enderami et al., 2017; Enderami et al., 2018a). These cells not only express markers of mature beta cells but also secrete insulin in response to glucose stimulation *in vitro* and *in vivo*. This strategy was applied to cells from T1DM patients, which obtained functional beta cells with no major differences compared with beta cells derived from normal iPSCs. This strategy has high efficiency for production of beta cells derived from stem cells, but the large numbers of growth factors and compounds make the procedure laborious. In this study, extracellular vesicles secreted by human beta cells were used to replace these growth factors and compounds. The obtained beta cells responded to glucose both *in vivo* and *in vitro*, and ameliorated hyperglycemia in STZ-induced diabetic mice.

Extracellular vesicles are secreted from host cells, which carry bioactive cargos and play major roles in immune responses, cancer development, tissue homeostasis, inflammation, angiogenesis, and stem cell differentiation (Raposo and Stoorvogel, 2013; Konala et al., 2016). Extracellular vesicles derived from dental pulp cells cultured under growth and odontogenic differentiation conditions induce odontogenic differentiation of human dental pulp stem cells and bone marrow derived stromal cells *in vitro* and *in vivo* (Huang et al., 2016). Exosomes derived from osteoblasts and adipocytes contain differentiation factors, including RNAs and proteins for osteoblast (RUNX2 and OSX) and adipocyte (C/EBPα and PPARγ) differentiation, which regulate the lineage specification of human mesenchymal stem cells (Narayanan et al., 2018). Additionally, several miRNAs have been identified in these exosomes, which promote the differentiation of human mesenchymal stem cells, including miR-34a, miR-27a and miR-22 for osteoblast differentiation and miR-143 and miR-375 for adipocyte differentiation. In our study, extracellular vesicles secreted from human pancreatic beta cells were used to promote the differentiation of iPSCs. Analyses of extracellular vesicles revealed the presence of some lineage miRNAs, including miR-16, Let-7, miR-212-3p, miR-132-3p, and miR-21-5p, and other miRNAs. MiR-212/132 play an important role in secretion of insulin from mature pancreatic beta cells (Malm et al., 2016; Mollet et al., 2016), but no reports have investigated regulation of the development and differentiation of pancreatic endocrine cells. In this study, miR-212/132 promoted differentiation of pancreatic beta cells by preventing translation of FBW7 to stabilize NGN3 expression. FBW7 is a ubiquitin E3 ligase substrate adaptor that targets many important oncoproteins for ubiquitin-dependent proteolysis, including c-Jun, cyclin E, c-Myc, and Notch. Emerging evidence has shown that FBW7 controls self-renewal, differentiation, survival, and multipotency in various stem cell types, including those of the hematopoietic and nervous systems, liver, and intestines (Hoeck et al., 2010; Wang et al., 2011; Sancho et al., 2013; Siu et al., 2014). FBW7 also acts as a master regulator of cell fate decisions in the pancreas and loss of FBW7 stabilizes NGN3 to promote adult pancreatic duct cell differentiation into endocrine cells (Sancho et al., 2014). NGN3 is an important regulator of endocrine cell formation during embryonic development. In iPSCs treated with miR-212/132 delivered by extracellular vesicles derived from beta cells, miR-212/132 stabilized NGN3 by targeting FBW7 to promote differentiation to beta cells. However, miR-212/132 also play an important role in the control of insulin secretion in human beta cells. We demonstrated that miR-212/132 inhibited FBW7 expression to promote the formation of i-Beta cells only, but the influence of miR-212/132 in insulin secretion and the mechanism underlying this in i-Beta cells are unclear. For the clinical application of i-Beta cell replacement therapy in T1DM, the mechanism underlying miR-212/132-regulated insulin secretion in i-Beta cells should be further investigated.

## Conclusion

**FIGURE 10:**
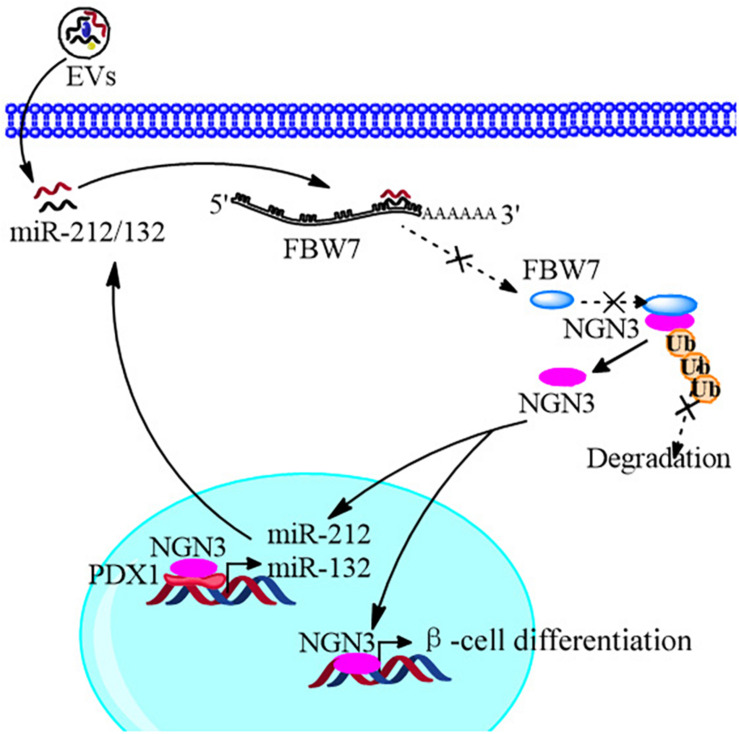
Schematic diagram of the EVs promoting β-cell differentiation from human pluripotent stem cells.

In conclusion, we improved the strategy of beta cell differentiation from iPSCs using miR-212/132-enriched extracellular vesicles and elucidated the important role of miR-212/132 in this process, which targeted FBW7 to stabilized NGN3 expression. Moreover, NGN3 bound to PDX1 to enhance transcription of endogenous miR-212/132 to form a positive regulatory circuit that maintained the functions of mature pancreatic beta cells (Figure 10). This study presents a new strategy to generate pancreatic beta cells from iPSCs *in vitro* and a potential source of pancreatic beta cells for transplantation therapy of type 1 diabetes mellitus.

## Methods

### Cell Culture

Human iPSCs were obtained as reported previously (Cui et al., 2020), which were reprogrammed by an inactivated Sendai virus from healthy female peripheral blood mononuclear cells, and cultured in mTesR1 complete medium (STEM CELL Technologies, BC, Canada) on diluted Matrigel (1:100, Corning, NY, United States). The cells were subcultured every 4 days by incubation with 0.5 mM EDTA (Thermo Fisher Scientific, United States) at 37°C for 3 min. Human beta cells were cultured in high glucose DMEM supplemented with 10% serum replacement. At 80% confluence, the cells were trypsinized with 0.25% trypsin and 0.01% EDTA (Gibco).

### EV Isolation and Characterization

A culture supernatant was obtained from human beta cells cultured in serum-free medium. Cell debris was removed by centrifugation at 2,000 *g* for 5 min and the culture supernatant was concentrated using centrifugal filter units (Millipore, MA, United States) by centrifugation at 2,000 *g* for 20 min. EVs in the supernatant were isolated by size exclusion chromatography using a qEV original size exclusion column (IZON science, Oxford, United Kingdom). The sample was added to the column and seven to nine 0.5 ml fractions were obtained and concentrated. The EVs were characterized by electron microscopy, nanoparticle tracking analysis (NanoSight LM10), and immunoblotting. Protein concentrations of EV preparations were measured by the Bradford method (CWBio, Beijing, China). Human iPSCs were incubated with 15 μg/mL purified EVs from 1 × 10^5^ cells to investigate their role in the formation of i-Beta cells.

### i-Beta Cell Differentiation and Characterization

A four-stage procedure (Figure 1A) was used to generate i-Beta cells *in vitro* as described previously (Pagliuca et al., 2014) with some improvements. The basal medium was used in the four-stage procedure, which contained DMEM/F12 (Invitrogen, CA, United States), B27 (1:100, Invitrogen), N2 (1:200, Invitrogen), 0.2% BSA (Sigma, MO, United States), ITS-X (1:100, Invitrogen), and 0.25 mM vitamin C (Sigma). In stage I, dissociated iPSCs were incubated in basal medium supplemented with 100 ng/ml activin A (PeproTech, United States) and 3 μM Chir-99021 (Selleck, United States) for 3 days. In stage II, the cells were cultured in basal medium supplemented with 50 ng/ml KGF (PeproTech) for 3 days. In stage III, the cells were cultured in basal medium supplemented with 2 μM retinoic acid (Sigma-Aldrich), 50 ng/ml KGF, 500 nM PdBU (MCE), 200 nM LDN193189 (MCE), and 0.25 μM Sant1 (MCE) for 4 days. In stage IV, the differentiated iPSCs were incubated in basal medium supplemented with 15 μg/mL purified EVs from 1 × 10^5^ cells for 15 days. EVs were renewed every 3 days. To examine the functions of pancreatic beta cells, i-Beta cells were assessed for insulin secretion in low glucose (2 mM) and high glucose (20 mM) in accordance with a previous report (Pagliuca et al., 2014).

To investigate the role of miRNAs derived from extracellular vesicles in the formation of i-Beta cells from iPSCs, the inhibitors of miR-21-5p, miR-29a-3p, miR-7b-5p, Let-7f, miR-212-3p, and miR-132-3p were synthesized and transfected into iPSCs using Lipofectamine 3000 (Invitrogen) combining with extracellular vesicle incubation at the start of stage IV. To demonstrate the function of miR-212/132 in the formation of i-Beta cells, mimics of miR-212-3p and miR-132-3p were synthesized, and singly transfected into iPSCs at the start of stage IV without EV incubation. Then, plasmids of SOX6, PDE7B, and FBW7, the targets of miR-212/132, were constructed and transfected into iPSCs using Lipofectamine 3000 (Invitrogen) combining with extracellular vesicle incubation at the start of stage IV. The expression of insulin-positive cells after the different treatments was analyzed using flow cytometry.

### Measurement of Extracellular Ca^2+^ Influxes in i-Beta Cells and iPSCs

i-Beta cells were seeded in plates coated with Matrigel and incubated with 50 μM Ca^2+^-sensitive fluorescent probe Fluo4-AM (Life Technologies) at 37°C for 30 min and then washed with PBS. The cells were then placed in the cell station of a TE2000 confocal microscope (Nikon) for high resolution time series imaging. Image acquisition started with 30 s of incubation in a 2 mM glucose solution, followed by 30 s of incubation in a 20 mM glucose solution, and then sequential 30 s of incubation in 2 and 20 mM glucose solutions repeated twice. Images were analyzed using ImageJ software.

The non-invasive micro-test technique (NMT, BIO-001A, Younger Sci. & Tech. Co., Amherst, MA, United States) was used to directly measure influxes of Ca^2+^ into i-Beta cells (Kuhtreiber and Jaffe, 1990). The electrode was controlled to move with an excursion of 10 μm at a programmable frequency in the range of 0.3–0.5 Hz. The cells were incubated in bathing buffer (Xuyue, Beijing, China) containing 2 mM glucose for 50 s and then the concentration of glucose was increased to 20 mM. The signals of Ca^2+^ fluxes were collected by micropipettes (2–4-μm aperture, XYPG120-2, Xuyue Science and Technology Co., Ltd., Beijing, China). Ca^2+^ fluxes were calculated by Fick’s law of diffusion: J = −D (dC/dX), where J represents the Ca2 + flux (μmol/cm^2^ per second), D is the ion diffusion constant in a particular solution and temperature (cm^2^/s), dC is the Ca^2+^ concentration difference (1 × 10^–3^ mol/L), and dX is the 10-μm excursion over which the electrode moved in the detection. Data and image acquisition, preliminary processing, control of the three-dimensional electrode positioner, and stepper motor-controlled fine focus of the microscope stage were performed using ASET software (Zhang Z. Y. et al., 2009; Li et al., 2012).

### Electron Microscopy

Extracellular vesicles were dropped onto the copper grid for 2 min and covered by uranyl acetate for 2 min. After air drying for 10 min, the shape of the extracellular vesicles was observed by transmission electron microscopy (TEM) at 100 kV. To analyze granular ultrastructures, insulin granules were observed by TEM after fixation, dehydration, permeabilization, and sectioning of i-Beta cells collected after various treatments. Images from at least two independent groups and at least three independent different batches for each treatment were recorded. ImageJ software was used to analyze and quantify the images.

### Nanosight Analysis

The size distribution of extracellular vesicles was measured by Nanoparticle Tracking Analysis using the NanoSight system (NanoSight, United Kingdom). The device measures the Brownian motion of particles whose speed of motion, or diffusion coefficient (Dt), is related to particle size through the Stokes–Einstein equation.

### qPCR

EV miRNAs were isolated with an EV extraction and RNA isolation kit (Rengen Biosciences, Shenyang, China). cDNA was synthesized using a High Capacity RNA-to-cDNA Kit (Tiangen, Beijing, China). Primers for the external control, miR-16, Let-7f, miR-21-5p, miR-29-3p, miR-25-3p, miR-146a-5p, miR-7b-5p, miR-212-3p, miR-132-3p, and Let-7a were purchased from Tiangen. qPCR was performed using SYBR PCR Mix (SYBR Green; Tiangen) in a Light Cycler 480 PCR system (Roche, USA). An external control (Tiangen, Beijing, China) was used for normalization. For mRNA, total RNA was extracted using Trizol reagent and reverse transcribed with sequence-specific primers. PCR was performed using an RNA PCR kit version 3.0 (Takara, China).

Gene expression was detected using the following primers. OCT4, F: GACAGGGGGAGGGGAGGAGCTAGG, R: CTTCCCTCCAACCAGTTGCCCCAAAC; NANOG, F: CAGC CCCGATTCTTCCACCAGTCCC, R: CGGAAGATTCCCAGT CGGGTTCACC; FOXA2, F: CAAGGGCCAGAGTTCCACAA, R: CCTGCAACCAGACAGGGTAT; NGN3, F: TTTTCTCCTT TGGGGCTGGG, R: AGGCGTCATCCTTTCTACCG; PDX1, F: CAGTTGAATGGGGCGGCAA, R: CAAGGTGGAGT GCTGTAGGAG; NKX6.1, F: TGGCCTGTACCCCTCATCAA, R: GAATAGGCCAAACGAGCCCT; NKX2.5, F: AGAC GGGGTTTTCGGTCAAG, R: GACCGTGCAGGGAGTAC TGAA; Insulin, F: GCAGCCTTTGTGAACCAACAC, R: CCCCGCACACTAGGTAGAGA. Each experiment was performed in duplicate in 96-well plates and repeated three times.

### Luciferase Reporter Assay

To predict miR-212/132-binding sites in the 3′ UTR of FBW7, firefly luciferase reporter vectors were constructed with the psiCHECK2 plasmid and FBW7 3′ UTR. A mutation at the nucleotide position of the miRNA seed sequence in the FBW7 3′ UTR was generated using a Fast Site-Directed Mutagenesis Kit (Tiangen) in accordance with the manufacturer’s instructions. Lipofectamine 3000 was used to transfect HEK293T cells with a mixture of the firefly luciferase reporter plasmid, miRNA precursor or control precursor, and Renilla reniformis luciferase-encoding plasmid. Cells transfected without precursors served as controls for normalization.

To detect promoter activity of miR-212/132, serial mutated fragments of the promoter region were inserted into pGL3.0-Luc. The plasmids and Renilla luciferase expression vector (pRL-40) were cotransfected into HEK293T cells. To examine the effect of transcription factors on promoter activity, PDX1 or NGN3 expression plasmids were mixed with pRL-40 and promoter luciferase reporter constructs with normal or mutant binding sites and cotransfected into HEK293T cells. Luciferase activity was measured at 48 h post-transfection using a dual-luciferase assay system. All detections were repeated independently at least three times.

### Coimmunoprecipitation

Flag-NGN3, Myc-FBW7, and HA-Ub plasmids were constructed and cotransfected into HEK293T cells. The cells were washed once with modified Dulbecco’s PBS, harvested, and lysed in 1 mL lysis buffer. The lysates were centrifuged and anti-Flag antibodies (1 μg) were added to 500 μL of the lysate. Immunoprecipitation was conducted with a Co-Immunoprecipitation Kit (Wanlei, Shenayng, China) in accordance with the manufacturer’s instructions and the products were analyzed by western blotting.

### Western Blotting

Cells were lysed in lysis buffer containing a protease inhibitor. The concentration of the protein extract was measured using a BCA assay and then the sample was subjected to SDS-PAGE, followed by transfer onto a PVDF membrane. Primary antibodies (Flag, 1:1000; Myc, 1:1000; HA, 1:1000; PDX1, 1:500; NGN3, 1:600; β-actin, 1:5000) and HRP-labeled secondary antibodies (1:5000) were purchased from Abcam (Cambridge, MA, United States). Proteins were visualized with Pierce ECL western blotting substrate for HRP. β-Actin was used as an internal control.

### miRNA Sequencing

Total RNA was size fractionated by 15% polyacrylamide gel electrophoresis and the 16–30 nt fraction was collected. The 5′ and 3′ RNA adaptors were ligated to the RNA and RNAs of 64–99 nt were isolated by gel elution and ethanol precipitation. Polymerase chain reaction (PCR) products were purified and small RNA libraries were sequenced using an Illumina Genome Analyzer. Sequencing was conducted at Shanghai Sinomics (Shanghai, China). Sequence files (fastq) were mapped to the reference miRBase21 using Bowtie. Gene abundance was expressed as counts of exon model per million mapped reads (CPM). Differential expression analysis for miRNAs was performed using DESeq software. Differentially expressed miRNAs with | log2(FC)| value>1 and *p* value < 0.05, considered as significantly modulated, were retained for further analysis.

### Flow Cytometry

To determine the level of insulin expression in iPSCs after different treatments, the cells were analyzed using an anti-insulin antibody (1:200, Abcam, United States) in an FC500 flow cytometer (Beckman Coulter, United States). Briefly, cells were collected, blocked, and labeled with the FITC-conjugated antibody against insulin in accordance with the manufacturer’s instructions. The data were analyzed with CXP software (Beckman Coulter). iPSCs were used as a negative control. Mean fluorescence intensity was determined after subtracting the value of negative control (iPSCs).

### Transplantation Studies

Transplantation studies were performed in accordance with previous reports (Shi et al., 2005; Pagliuca et al., 2014). Human beta cells, iPSCs, and i-Beta cells (3 × 10^6^ cells per animal) were transplanted into the renal capsule of immunodeficiency SCID mice at 6 weeks of age under aseptic conditions. After 2 weeks, mice were intraperitoneally injected with 2 g of glucose/kg body weight, and serum was obtained from snipped tails after 0 and 30 min to determine the human insulin levels with an insulin ELISA kit. Cell grafts were collected from the kidneys, fixed in 4% PFA overnight, embedded in paraffin, sectioned, and analyzed with insulin and glucagon antibody by IHC. Diabetic mice were obtained after streptozotocin treatment at 100 mg/kg for 3 days. When the blood glucose levels of the diabetic mice rose above 13.9 mM, 3 × 10^6^ human beta cells, iPSCs, or i-Beta cells were transplanted into the renal capsule. Blood glucose was measured from snipped tails every 6 days after transplantation.

### Statistical Analysis

All experiments were performed at least three times independently and in triplicate. Results are presented as the mean ± standard deviation (SD). Differences were assessed by Student’s *t*-test. *p* < 0.05 was considered statistically significant.

## Data Availability Statement

The miRNA sequencing data generated in this study have been deposited on the Gene Expression Omnibus (GEO) repository (accession numbers:GSE162801).

## Ethics Statement

The animal study was reviewed and approved by the Committee on the Ethics of Animal Experiments of Jining Medical University (License ID: 2017-JZ-003).

## Author Contributions

CB performed cell culture, transfection, western blotting, FCM, and drafted the manuscript. QR and HL prepared the mouse model. HL analyzed blood biochemical values of mice. YG performed exosome isolation and biological analysis, RNA isolation, and real-time PCR, designed the experiments and reviewed the manuscript. XL analyzed data and reviewed the manuscript. WG participated in its design and coordination. All authors contributed to the article and approved the submitted version.

## Conflict of Interest

The authors declare that the research was conducted in the absence of any commercial or financial relationships that could be construed as a potential conflict of interest.
