# Corrigendum: miR-212/132-enriched extracellular vesicles promote differentiation of induced pluripotent stem cells into pancreatic beta cells

**DOI:** 10.3389/fcell.2023.1258178

**Published:** 2023-08-01

**Authors:** Chunyu Bai, Qiwei Ren, Haifeng Liu, Xiangchen Li, Weijun Guan, Yuhua Gao

**Affiliations:** ^1^ Institute of Precision Medicine, Jining Medical University, Jining, China; ^2^ Institute of Animal Sciences, Chinese Academy of Agricultural Sciences, Beijing, China; ^3^ College of Basic Medicine, Jining Medical University, Jining, China; ^4^ Department of Laboratory Medicine, Affiliated Hospital of Jining Medical University, Jining, China; ^5^ College of Animal Science and Technology, College of Veterinary Medicine, Zhejiang A&F University, Lin’an, China

**Keywords:** iPSCs, beta cells, differentiation, extracellular vesicles, miRNAs

In the published article, there was an error in Figure 2 as published. The image of i-Beta cells was lost. The corrected Figure 2 appear below.

**FIGURE 2:**
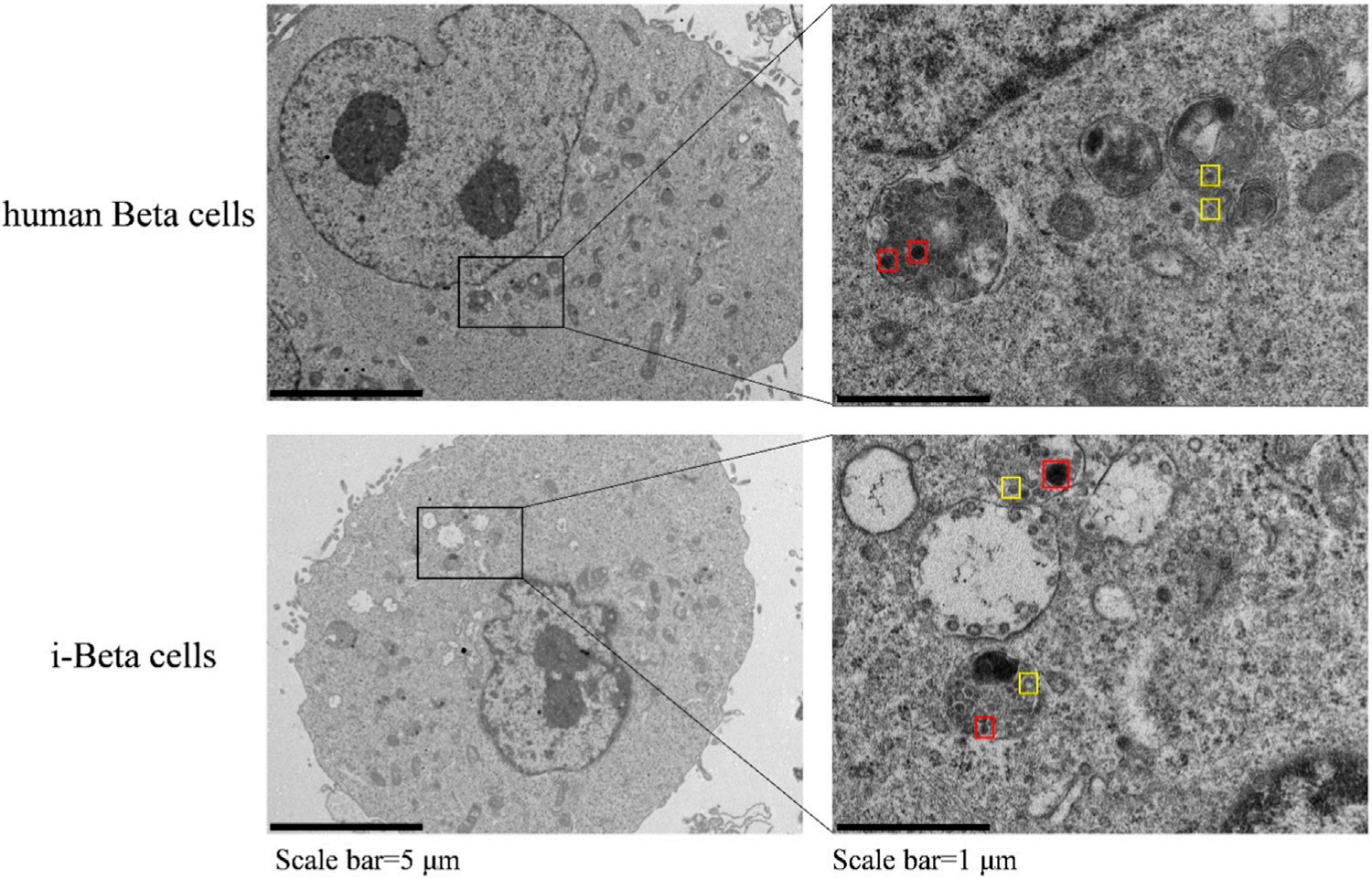
Electron microscopy of granules in sectioned cells with representative crystallized insulin granules (red) and early insulin granules (yellow) in human beta cells and i-Beta cells. Left panel shows electron microscopy images of granules in human beta cells (top image) and i-Beta cells (bottom image). Right panel shows a box and whisker plot of the numbe of insulin granules per cell (*n* = 20, Scale bar = 1 µm).

The authors apologize for this error and state that this does not change the scientific conclusions of the article in any way. The original article has been updated.

