# Supplementary material for: miR-212/132-Enriched Extracellular Vesicles Promote Differentiation of Induced Pluripotent Stem Cells Into Pancreatic Beta Cells
Source: Front Cell Dev Biol. 2021 May 13;9:673231. doi: 10.3389/fcell.2021.673231 (PMC8155495; doi:10.3389/fcell.2021.673231)
Supplement: Supplementary file 1 [file Data_Sheet_1.PDF]

Table S1 miRNA profile in extracellular vesicles derived from beta cells

ID	MIR	ID	MIR	ID	MIR	ID	MIR	ID	MIR
hsa-let-7a-2-3p	MIMAT0010195	hsa-miR-222-3p	MIMAT0000279	hsa-miR-411-5p	MIMAT0003329	hsa-miR-561-3p	MIMAT0003225	hsa-miR-943	MIMAT0004986
hsa-let-7a-3p	MIMAT0004481	hsa-miR-222-5p	MIMAT0004569	hsa-miR-412-3p	MIMAT0002170	hsa-miR-561-5p	MIMAT0022706	hsa-miR-95-3p	MIMAT0000094
hsa-let-7a-5p	MIMAT0000062	hsa-miR-224-5p	MIMAT0000281	hsa-miR-412-5p	MIMAT0026557	hsa-miR-5684	MIMAT0022473	hsa-miR-96-5p	MIMAT0000095
hsa-let-7b-3p	MIMAT0004482	hsa-miR-2276-3p	MIMAT0011775	hsa-miR-421	MIMAT0003339	hsa-miR-5690	MIMAT0022482	hsa-miR-98-3p	MIMAT0022842
hsa-let-7b-5p	MIMAT0000063	hsa-miR-2277-3p	MIMAT0011777	hsa-miR-423-3p	MIMAT0001340	hsa-miR-5697	MIMAT0022490	hsa-miR-98-5p	MIMAT0000096
hsa-let-7c-5p	MIMAT0000064	hsa-miR-2277-5p	MIMAT0017352	hsa-miR-423-5p	MIMAT0004748	hsa-miR-5698	MIMAT0022491	hsa-miR-99a-3p	MIMAT0004511
hsa-let-7d-3p	MIMAT0004484	hsa-miR-2278	MIMAT0011778	hsa-miR-424-3p	MIMAT0004749	hsa-miR-5699-3p	MIMAT0022492	hsa-miR-99a-5p	MIMAT0000097
hsa-let-7d-5p	MIMAT0000065	hsa-miR-2355-3p	MIMAT0017950	hsa-miR-424-5p	MIMAT0001341	hsa-miR-5699-5p	MIMAT0027103	hsa-miR-99b-3p	MIMAT0004678
hsa-	MIM	hsa-	MIMAT	hsa-	MIM	hsa-	MIMAT	hsa-	MIM

let-7e-3p	AT0004485	miR-23a-3p	0000078	miR-425-3p	AT0001343	miR-570-3p	0003235	miR-99b-5p	AT0000689
hsa-let-7e-5p	MIMAT0000066	hsa-miR-23a-5p	MIMAT0004496	hsa-miR-425-5p	MIMAT0003393	hsa-miR-5701	MIMAT0022494		
hsa-let-7f-1-3p	MIMAT0004486	hsa-miR-23b-3p	MIMAT0000418	hsa-miR-4254	MIMAT0016884	hsa-miR-574-3p	MIMAT0003239		
hsa-let-7f-2-3p	MIMAT0004487	hsa-miR-23b-5p	MIMAT0004587	hsa-miR-4268	MIMAT0016896	hsa-miR-574-5p	MIMAT0004795		
hsa-let-7f-5p	MIMAT0000067	hsa-miR-23c	MIMAT0018000	hsa-miR-4284	MIMAT0016915	hsa-miR-575	MIMAT0003240		
hsa-let-7g-3p	MIMAT0004584	hsa-miR-24-1-5p	MIMAT0000079	hsa-miR-4286	MIMAT0016916	hsa-miR-576-3p	MIMAT0004796		
hsa-let-7g-5p	MIMAT0000414	hsa-miR-24-2-5p	MIMAT0004497	hsa-miR-429	MIMAT0001536	hsa-miR-576-5p	MIMAT0003241		
hsa-let-7i-3p	MIMAT0004585	hsa-miR-24-3p	MIMAT0000080	hsa-miR-4298	MIMAT0016852	hsa-miR-5787	MIMAT0023252		
hsa-let-7i-5p	MIMAT0000415	hsa-miR-25-3p	MIMAT0000081	hsa-miR-4306	MIMAT0016858	hsa-miR-579-3p	MIMAT0003244		
hsa-miR-1-3p	MIMAT0000416	hsa-miR-25-5p	MIMAT0004498	hsa-miR-431-3p	MIMAT0004757	hsa-miR-579-5p	MIMAT0026616		
hsa-	MIM	hsa-	MIMAT	hsa-	MIM	hsa-	MIMAT		

miR-100-3p	AT0004512	miR-2682-3p	0013518	miR-431-5p	AT0001625	miR-580-3p	0003245		
hsa-miR-100-5p	MIMAT0000098	hsa-miR-2682-5p	MIMAT0013517	hsa-miR-4315	MIMAT0016866	hsa-miR-582-3p	MIMAT0004797		
hsa-miR-101-3p	MIMAT0000099	hsa-miR-26a-1-3p	MIMAT0004499	hsa-miR-432-5p	MIMAT0002814	hsa-miR-582-5p	MIMAT0003247		
hsa-miR-103a-3p	MIMAT0000101	hsa-miR-26a-2-3p	MIMAT0004681	hsa-miR-4326	MIMAT0016888	hsa-miR-583	MIMAT0003248		
hsa-miR-105-5p	MIMAT0000102	hsa-miR-26a-5p	MIMAT0000082	hsa-miR-433-3p	MIMAT0001627	hsa-miR-584-3p	MIMAT0022708		
hsa-miR-106a-5p	MIMAT0000103	hsa-miR-26b-3p	MIMAT0004500	hsa-miR-433-5p	MIMAT0026554	hsa-miR-584-5p	MIMAT0003249		
hsa-miR-106b-3p	MIMAT0004672	hsa-miR-26b-5p	MIMAT0000083	hsa-miR-4417	MIMAT0018929	hsa-miR-585-5p	MIMAT0026618		
hsa-miR-106b-5p	MIMAT0000680	hsa-miR-27a-3p	MIMAT0000084	hsa-miR-4419b	MIMAT0019034	hsa-miR-586	MIMAT0003252		
hsa-miR-107	MIMAT0000104	hsa-miR-27a-5p	MIMAT0004501	hsa-miR-4421	MIMAT0018934	hsa-miR-589-3p	MIMAT0003256		

hsa-miR-10a-3p	MIMAT0004555	hsa-miR-27b-3p	MIMAT0000419	hsa-miR-4425	MIMAT0018940	hsa-miR-589-5p	MIMAT0004799		
hsa-miR-10a-5p	MIMAT0000253	hsa-miR-27b-5p	MIMAT0004588	hsa-miR-4428	MIMAT0018943	hsa-miR-590-3p	MIMAT0004801		
hsa-miR-10b-3p	MIMAT0004556	hsa-miR-28-3p	MIMAT0004502	hsa-miR-4429	MIMAT0018944	hsa-miR-590-5p	MIMAT0003258		
hsa-miR-10b-5p	MIMAT0000254	hsa-miR-28-5p	MIMAT0000085	hsa-miR-4435	MIMAT0018951	hsa-miR-597-5p	MIMAT0003265		
hsa-miR-1180-3p	MIMAT0005825	hsa-miR-296-3p	MIMAT0004679	hsa-miR-4440	MIMAT0018958	hsa-miR-598-3p	MIMAT0003266		
hsa-miR-1185-1-3p	MIMAT0022838	hsa-miR-296-5p	MIMAT0000690	hsa-miR-4443	MIMAT0018961	hsa-miR-602	MIMAT0003270		
hsa-miR-1185-2-3p	MIMAT0022713	hsa-miR-298	MIMAT0004901	hsa-miR-4445-3p	MIMAT0018964	hsa-miR-605-3p	MIMAT0026621		
hsa-miR-1185-5p	MIMAT0005798	hsa-miR-299-3p	MIMAT0000687	hsa-miR-4445-5p	MIMAT0018963	hsa-miR-6087	MIMAT0023712		
hsa-miR-1193	MIMAT0015049	hsa-miR-299-5p	MIMAT0002890	hsa-miR-4448	MIMAT0018967	hsa-miR-6089	MIMAT0023714		
hsa-	MIM	hsa-	MIMAT	hsa-	MIM	hsa-	MIMAT		

miR-1197	AT0005955	miR-29a-3p	0000086	miR-4449	AT0018968	miR-610	0003278		
hsa-miR-1200	MIMAT0005863	hsa-miR-29a-5p	MIMAT0004503	hsa-miR-4454	MIMAT0018976	hsa-miR-612	MIMAT0003280		
hsa-miR-1204	MIMAT0005868	hsa-miR-29b-1-5p	MIMAT0004514	hsa-miR-4457	MIMAT0018979	hsa-miR-6125	MIMAT0024598		
hsa-miR-1207-5p	MIMAT0005871	hsa-miR-29b-2-5p	MIMAT0004515	hsa-miR-4458	MIMAT0018980	hsa-miR-6127	MIMAT0024610		
hsa-miR-122-5p	MIMAT0000421	hsa-miR-29b-3p	MIMAT0000100	hsa-miR-4459	MIMAT0018981	hsa-miR-615-3p	MIMAT0003283		
hsa-miR-1224-3p	MIMAT0005459	hsa-miR-29c-3p	MIMAT0000681	hsa-miR-4461	MIMAT0018983	hsa-miR-615-5p	MIMAT0004804		
hsa-miR-1224-5p	MIMAT0005458	hsa-miR-29c-5p	MIMAT0004673	hsa-miR-4466	MIMAT0018993	hsa-miR-616-3p	MIMAT0004805		
hsa-miR-1226-3p	MIMAT0005577	hsa-miR-301a-3p	MIMAT0000688	hsa-miR-4467	MIMAT0018994	hsa-miR-616-5p	MIMAT0003284		
hsa-miR-1226-5p	MIMAT0005576	hsa-miR-301a-5p	MIMAT0022696	hsa-miR-4468	MIMAT0018995	hsa-miR-6165	MIMAT0024782		

hsa-miR-1229-3p	MIMAT0005584	hsa-miR-301b-3p	MIMAT0004958	hsa-miR-4474-3p	MIMAT0019001	hsa-miR-618	MIMAT0003287		
hsa-miR-1229-5p	MIMAT0022942	hsa-miR-301b-5p	MIMAT0032026	hsa-miR-4479	MIMAT0019011	hsa-miR-619-5p	MIMAT0026622		
hsa-miR-1234-3p	MIMAT0005589	hsa-miR-3064-5p	MIMAT0019864	hsa-miR-4482-3p	MIMAT0020958	hsa-miR-622	MIMAT0003291		
hsa-miR-1236-5p	MIMAT0022945	hsa-miR-3065-3p	MIMAT0015378	hsa-miR-4484	MIMAT0019018	hsa-miR-624-5p	MIMAT0003293		
hsa-miR-1244	MIMAT0005896	hsa-miR-3065-5p	MIMAT0015066	hsa-miR-4485-3p	MIMAT0019019	hsa-miR-625-3p	MIMAT0004808		
hsa-miR-1246	MIMAT0005898	hsa-miR-3074-3p	MIMAT0015027	hsa-miR-4488	MIMAT0019022	hsa-miR-625-5p	MIMAT0003294		
hsa-miR-1248	MIMAT0005900	hsa-miR-3074-5p	MIMAT0019208	hsa-miR-4489	MIMAT0019023	hsa-miR-627-3p	MIMAT0026623		
hsa-miR-1249-3p	MIMAT0005901	hsa-miR-30a-3p	MIMAT0000088	hsa-miR-4492	MIMAT0019027	hsa-miR-627-5p	MIMAT0003296		
hsa-miR-1250-5p	MIMAT0005902	hsa-miR-30a-	MIMAT0000087	hsa-miR-4497	MIMAT0019032	hsa-miR-628-3p	MIMAT0003297		

		5p							
hsa-miR-1255a	MIMAT0005906	hsa-miR-30b-3p	MIMAT0004589	hsa-miR-449a	MIMAT0001541	hsa-miR-628-5p	MIMAT0004809		
hsa-miR-1255b-5p	MIMAT0005945	hsa-miR-30b-5p	MIMAT0000420	hsa-miR-449b-5p	MIMAT0003327	hsa-miR-629-3p	MIMAT0003298		
hsa-miR-125a-3p	MIMAT0004602	hsa-miR-30c-1-3p	MIMAT0004674	hsa-miR-449c-5p	MIMAT0010251	hsa-miR-629-5p	MIMAT0004810		
hsa-miR-125a-5p	MIMAT0000443	hsa-miR-30c-2-3p	MIMAT0004550	hsa-miR-4500	MIMAT0019036	hsa-miR-636	MIMAT0003306		
hsa-miR-125b-1-3p	MIMAT0004592	hsa-miR-30c-5p	MIMAT0000244	hsa-miR-4504	MIMAT0019040	hsa-miR-639	MIMAT0003309		
hsa-miR-125b-2-3p	MIMAT0004603	hsa-miR-30d-3p	MIMAT0004551	hsa-miR-4508	MIMAT0019045	hsa-miR-641	MIMAT0003311		
hsa-miR-125b-5p	MIMAT0000423	hsa-miR-30d-5p	MIMAT0000245	hsa-miR-450a-1-3p	MIMAT0022700	hsa-miR-642a-5p	MIMAT0003312		
hsa-miR-126-3p	MIMAT0000445	hsa-miR-30e-3p	MIMAT0000693	hsa-miR-450a-2-3p	MIMAT0031074	hsa-miR-642b-3p	MIMAT0018444		
hsa-miR-126-	MIMAT0000444	hsa-miR-	MIMAT0000692	hsa-miR-450a-	MIMAT0001545	hsa-miR-643	MIMAT0003313		

5p		30e-5p		5p					
hsa-miR-1260a	MIMAT0005911	hsa-miR-31-3p	MIMAT0004504	hsa-miR-450b-3p	MIMAT0004910	hsa-miR-6500-3p	MIMAT0025455		
hsa-miR-1260b	MIMAT0015041	hsa-miR-31-5p	MIMAT0000089	hsa-miR-450b-5p	MIMAT0004909	hsa-miR-6501-5p	MIMAT0025458		
hsa-miR-1262	MIMAT0005914	hsa-miR-3115	MIMAT0014977	hsa-miR-4511	MIMAT0019048	hsa-miR-6505-3p	MIMAT0025467		
hsa-miR-1267	MIMAT0005921	hsa-miR-3116	MIMAT0014978	hsa-miR-4512	MIMAT0019049	hsa-miR-6505-5p	MIMAT0025466		
hsa-miR-1268a	MIMAT0005922	hsa-miR-3117-3p	MIMAT0014979	hsa-miR-4514	MIMAT0019051	hsa-miR-6506-3p	MIMAT0025469		
hsa-miR-1268b	MIMAT0018925	hsa-miR-3124-3p	MIMAT0019200	hsa-miR-4516	MIMAT0019053	hsa-miR-6506-5p	MIMAT0025468		
hsa-miR-1269a	MIMAT0005923	hsa-miR-3124-5p	MIMAT0014986	hsa-miR-452-5p	MIMAT0001635	hsa-miR-6508-3p	MIMAT0025473		
hsa-miR-127-3p	MIMAT0000446	hsa-miR-3126-3p	MIMAT0015377	hsa-miR-4521	MIMAT0019058	hsa-miR-651-5p	MIMAT0003321		
hsa-miR-127-5p	MIMAT0004604	hsa-miR-312	MIMAT0014989	hsa-miR-4523	MIMAT0019061	hsa-miR-6510-5p	MIMAT0025476		

		6-5p							
hsa-miR-1271-5p	MIMAT0005796	hsa-miR-3127-3p	MIMAT0019201	hsa-miR-4524a-3p	MIMAT0019063	hsa-miR-6511a-3p	MIMAT0025479		
hsa-miR-1273a	MIMAT0005926	hsa-miR-3127-5p	MIMAT0014990	hsa-miR-4532	MIMAT0019071	hsa-miR-6511b-3p	MIMAT0025848		
hsa-miR-1273c	MIMAT0015017	hsa-miR-3128	MIMAT0014991	hsa-miR-4535	MIMAT0019075	hsa-miR-6511b-5p	MIMAT0025847		
hsa-miR-1273d	MIMAT0015090	hsa-miR-3129-3p	MIMAT0019202	hsa-miR-4539	MIMAT0019082	hsa-miR-6512-3p	MIMAT0025481		
hsa-miR-1273e	MIMAT0018079	hsa-miR-3129-5p	MIMAT0014992	hsa-miR-454-3p	MIMAT0003885	hsa-miR-6514-5p	MIMAT0025484		
hsa-miR-1273f	MIMAT0020601	hsa-miR-3130-3p	MIMAT0014994	hsa-miR-454-5p	MIMAT0003884	hsa-miR-6515-5p	MIMAT0025486		
hsa-miR-1273g-3p	MIMAT0022742	hsa-miR-3131	MIMAT0014996	hsa-miR-455-3p	MIMAT0004784	hsa-miR-652-3p	MIMAT0003322		
hsa-miR-1273h-5p	MIMAT0030415	hsa-miR-3132	MIMAT0014997	hsa-miR-455-5p	MIMAT0003150	hsa-miR-652-5p	MIMAT0022709		
hsa-miR-1275	MIMAT0005929	hsa-miR-	MIMAT0014998	hsa-miR-4636	MIMAT0019693	hsa-miR-654-	MIMAT0004814		

		3133				3p			
hsa-miR-1276	MIMAT0005930	hsa-miR-3135b	MIMAT0018985	hsa-miR-4638-3p	MIMAT0019696	hsa-miR-654-5p	MIMAT0003330		
hsa-miR-1277-3p	MIMAT0005933	hsa-miR-3136-5p	MIMAT0015003	hsa-miR-4639-5p	MIMAT0019697	hsa-miR-655-3p	MIMAT0003331		
hsa-miR-1277-5p	MIMAT0022724	hsa-miR-3138	MIMAT0015006	hsa-miR-4640-5p	MIMAT0019699	hsa-miR-655-5p	MIMAT0026626		
hsa-miR-1278	MIMAT0005936	hsa-miR-3140-3p	MIMAT0015008	hsa-miR-4645-3p	MIMAT0019706	hsa-miR-656-3p	MIMAT0003332		
hsa-miR-128-1-5p	MIMAT0026477	hsa-miR-3141	MIMAT0015010	hsa-miR-4646-3p	MIMAT0019708	hsa-miR-656-5p	MIMAT0026627		
hsa-miR-128-3p	MIMAT0000424	hsa-miR-3143	MIMAT0015012	hsa-miR-4646-5p	MIMAT0019707	hsa-miR-659-3p	MIMAT0003337		
hsa-miR-1284	MIMAT0005941	hsa-miR-3144-3p	MIMAT0015015	hsa-miR-4653-3p	MIMAT0019719	hsa-miR-660-3p	MIMAT0022711		
hsa-miR-1285-3p	MIMAT0005876	hsa-miR-3144-5p	MIMAT0015014	hsa-miR-4655-3p	MIMAT0019722	hsa-miR-660-5p	MIMAT0003338		
hsa-miR-	MIMAT00	hsa-miR	MIMAT0015016	hsa-miR-	MIMAT001	hsa-miR-	MIMAT0003326		

1285-5p	22719	-3145-3p		4655-5p	9721	663a			
hsa-miR-1286	MIMAT0005877	hsa-miR-3146	MIMAT0015018	hsa-miR-4658	MIMAT0019725	hsa-miR-663b	MIMAT0005867		
hsa-miR-1287-5p	MIMAT0005878	hsa-miR-3148	MIMAT0015021	hsa-miR-4659a-3p	MIMAT0019727	hsa-miR-664a-3p	MIMAT0005949		
hsa-miR-1289	MIMAT0005879	hsa-miR-3149	MIMAT0015022	hsa-miR-4659a-5p	MIMAT0019726	hsa-miR-664a-5p	MIMAT0005948		
hsa-miR-129-1-3p	MIMAT0004548	hsa-miR-3150a-3p	MIMAT0015023	hsa-miR-4660	MIMAT0019728	hsa-miR-664b-3p	MIMAT0022272		
hsa-miR-129-2-3p	MIMAT0004605	hsa-miR-3150a-5p	MIMAT0019206	hsa-miR-4661-3p	MIMAT0019730	hsa-miR-664b-5p	MIMAT0022271		
hsa-miR-129-5p	MIMAT0000242	hsa-miR-3150b-5p	MIMAT0019226	hsa-miR-4662a-5p	MIMAT0019731	hsa-miR-665	MIMAT0004952		
hsa-miR-1290	MIMAT0005880	hsa-miR-3151-3p	MIMAT0027026	hsa-miR-4664-3p	MIMAT0019738	hsa-miR-668-3p	MIMAT0003881		
hsa-miR-1291	MIMAT0005881	hsa-miR-	MIMAT0015025	hsa-miR-4664-	MIMAT0019737	hsa-miR-671-	MIMAT0004819		

		3152-3p		5p		3p			
hsa-miR-1292-5p	MIMAT0005943	hsa-miR-3157-5p	MIMAT0015031	hsa-miR-4667-3p	MIMAT0019744	hsa-miR-671-5p	MIMAT0003880		
hsa-miR-1293	MIMAT0005883	hsa-miR-3158-3p	MIMAT0015032	hsa-miR-4667-5p	MIMAT0019743	hsa-miR-6716-3p	MIMAT0025845		
hsa-miR-1294	MIMAT0005884	hsa-miR-3160-3p	MIMAT0015034	hsa-miR-4669	MIMAT0019749	hsa-miR-6720-3p	MIMAT0025851		
hsa-miR-1296-5p	MIMAT0005794	hsa-miR-3161	MIMAT0015035	hsa-miR-4671-3p	MIMAT0019753	hsa-miR-6720-5p	MIMAT0027345		
hsa-miR-1297	MIMAT0005886	hsa-miR-3162-5p	MIMAT0015036	hsa-miR-4671-5p	MIMAT0019752	hsa-miR-6723-5p	MIMAT0025855		
hsa-miR-1299	MIMAT0005887	hsa-miR-3164	MIMAT0015038	hsa-miR-4676-3p	MIMAT0019759	hsa-miR-6726-5p	MIMAT0027353		
hsa-miR-1301-3p	MIMAT0005797	hsa-miR-3165	MIMAT0015039	hsa-miR-4677-3p	MIMAT0019761	hsa-miR-6727-5p	MIMAT0027355		
hsa-miR-1303	MIMAT0005891	hsa-miR-3170	MIMAT0015045	hsa-miR-4677-5p	MIMAT0019760	hsa-miR-6728-5p	MIMAT0027357		
hsa-miR-	MIMAT00	hsa-miR	MIMAT0019214	hsa-miR-	MIMAT001	hsa-miR-	MIMAT0027363		

1305	05893	-3173-5p		4683	9768	6731-5p			
hsa-miR-1306-5p	MIMAT0022726	hsa-miR-3174	MIMAT0015051	hsa-miR-4684-3p	MIMAT0019770	hsa-miR-6732-3p	MIMAT0027366		
hsa-miR-1307-3p	MIMAT0005951	hsa-miR-3176	MIMAT0015053	hsa-miR-4684-5p	MIMAT0019769	hsa-miR-6733-3p	MIMAT0027368		
hsa-miR-1307-5p	MIMAT0022727	hsa-miR-3177-5p	MIMAT0019215	hsa-miR-4685-3p	MIMAT0019772	hsa-miR-6733-5p	MIMAT0027367		
hsa-miR-130a-3p	MIMAT0000425	hsa-miR-3178	MIMAT0015055	hsa-miR-4687-3p	MIMAT0019775	hsa-miR-6734-3p	MIMAT0027370		
hsa-miR-130b-3p	MIMAT0000691	hsa-miR-3180	MIMAT0018178	hsa-miR-4689	MIMAT0019778	hsa-miR-6734-5p	MIMAT0027369		
hsa-miR-130b-5p	MIMAT0004680	hsa-miR-3180-3p	MIMAT0015058	hsa-miR-4690-3p	MIMAT0019780	hsa-miR-6735-5p	MIMAT0027371		
hsa-miR-132-3p	MIMAT0000426	hsa-miR-3180-5p	MIMAT0015057	hsa-miR-4691-3p	MIMAT0019782	hsa-miR-6740-5p	MIMAT0027381		
hsa-miR-132-5p	MIMAT0004594	hsa-miR-3182	MIMAT0015062	hsa-miR-4706	MIMAT0019806	hsa-miR-6741-5p	MIMAT0027383		
hsa-	MIM	hsa-	MIMAT	hsa-	MIM	hsa-	MIMAT		

miR-1321	AT0005952	miR-3183	0015063	miR-4712-5p	AT0019818	miR-6747-3p	0027395		
hsa-miR-133a-3p	MIMAT0000427	hsa-miR-3184-5p	MIMAT0015064	hsa-miR-4713-5p	MIMAT0019820	hsa-miR-6748-3p	MIMAT0027397		
hsa-miR-134-3p	MIMAT0026481	hsa-miR-3187-3p	MIMAT0015069	hsa-miR-4714-3p	MIMAT0019823	hsa-miR-675-5p	MIMAT0004284		
hsa-miR-134-5p	MIMAT0000447	hsa-miR-3188	MIMAT0015070	hsa-miR-4717-3p	MIMAT0019830	hsa-miR-6753-3p	MIMAT0027407		
hsa-miR-1343-3p	MIMAT0019776	hsa-miR-3190-3p	MIMAT0022839	hsa-miR-4722-5p	MIMAT0019836	hsa-miR-6753-5p	MIMAT0027406		
hsa-miR-135a-5p	MIMAT0000428	hsa-miR-3191-3p	MIMAT0015075	hsa-miR-4725-3p	MIMAT0019844	hsa-miR-6754-5p	MIMAT0027408		
hsa-miR-135b-3p	MIMAT0004698	hsa-miR-3191-5p	MIMAT0022732	hsa-miR-4728-3p	MIMAT0019850	hsa-miR-6755-5p	MIMAT0027410		
hsa-miR-135b-5p	MIMAT0000758	hsa-miR-3192-5p	MIMAT0015076	hsa-miR-4732-3p	MIMAT0019856	hsa-miR-6756-5p	MIMAT0027412		
hsa-miR-136-3p	MIMAT0004606	hsa-miR-3193	MIMAT0015077	hsa-miR-4733-3p	MIMAT0019858	hsa-miR-676-3p	MIMAT0018204		

hsa-miR-136-5p	MIMAT0000448	hsa-miR-3195	MIMAT0015079	hsa-miR-4738-3p	MIMAT0019867	hsa-miR-676-5p	MIMAT0018203		
hsa-miR-137	MIMAT0000429	hsa-miR-3196	MIMAT0015080	hsa-miR-4739	MIMAT0019868	hsa-miR-6760-3p	MIMAT0027421		
hsa-miR-138-1-3p	MIMAT0004607	hsa-miR-3198	MIMAT0015083	hsa-miR-4742-3p	MIMAT0019873	hsa-miR-6760-5p	MIMAT0027420		
hsa-miR-138-2-3p	MIMAT0004596	hsa-miR-3199	MIMAT0015084	hsa-miR-4742-5p	MIMAT0019872	hsa-miR-6763-3p	MIMAT0027427		
hsa-miR-138-5p	MIMAT0000430	hsa-miR-32-3p	MIMAT0004505	hsa-miR-4744	MIMAT0019875	hsa-miR-6763-5p	MIMAT0027426		
hsa-miR-139-3p	MIMAT0004552	hsa-miR-32-5p	MIMAT0000090	hsa-miR-4746-3p	MIMAT0019881	hsa-miR-6764-5p	MIMAT0027428		
hsa-miR-139-5p	MIMAT0000250	hsa-miR-3200-3p	MIMAT0015085	hsa-miR-4746-5p	MIMAT0019880	hsa-miR-6767-5p	MIMAT0027434		
hsa-miR-140-3p	MIMAT0004597	hsa-miR-320a	MIMAT0000510	hsa-miR-4747-5p	MIMAT0019882	hsa-miR-6768-5p	MIMAT0027436		
hsa-miR-140-5p	MIMAT0000431	hsa-miR-320b	MIMAT0005792	hsa-miR-4755-5p	MIMAT0019895	hsa-miR-6769a-5p	MIMAT0027438		
hsa-miR-	MIMAT00	hsa-miR	MIMAT0005793	hsa-miR-	MIMAT001	hsa-miR-	MIMAT0027621		

141-3p	00432	-320c		4761-3p	9909	6769b-3p			
hsa-miR-142-3p	MIMAT0000434	hsa-miR-320d	MIMAT0006764	hsa-miR-4763-3p	MIMAT0019913	hsa-miR-6772-3p	MIMAT0027445		
hsa-miR-142-5p	MIMAT0000433	hsa-miR-320e	MIMAT0015072	hsa-miR-4766-5p	MIMAT0019917	hsa-miR-6773-3p	MIMAT0027447		
hsa-miR-143-3p	MIMAT0000435	hsa-miR-323a-3p	MIMAT0000755	hsa-miR-4768-5p	MIMAT0019920	hsa-miR-6775-3p	MIMAT0027451		
hsa-miR-143-5p	MIMAT0004599	hsa-miR-323a-5p	MIMAT0004696	hsa-miR-4769-3p	MIMAT0019923	hsa-miR-6777-5p	MIMAT0027454		
hsa-miR-145-3p	MIMAT0004601	hsa-miR-323b-3p	MIMAT0015050	hsa-miR-4775	MIMAT0019931	hsa-miR-6778-5p	MIMAT0027456		
hsa-miR-145-5p	MIMAT0000437	hsa-miR-324-3p	MIMAT0000762	hsa-miR-4776-3p	MIMAT0019933	hsa-miR-6779-3p	MIMAT0027459		
hsa-miR-1468-5p	MIMAT0006789	hsa-miR-324-5p	MIMAT0000761	hsa-miR-4776-5p	MIMAT0019932	hsa-miR-6779-5p	MIMAT0027458		
hsa-miR-1469	MIMAT0007347	hsa-miR-326	MIMAT0000756	hsa-miR-4783-3p	MIMAT0019947	hsa-miR-6781-3p	MIMAT0027463		
hsa-miR-146a-3p	MIMAT0004608	hsa-miR-328-	MIMAT0000752	hsa-miR-4785	MIMAT0019949	hsa-miR-6781-5p	MIMAT0027462		

		3p							
hsa-miR-146a-5p	MIMAT0000449	hsa-miR-328-5p	MIMAT0026486	hsa-miR-4786-5p	MIMAT0019954	hsa-miR-6784-5p	MIMAT0027468		
hsa-miR-146b-3p	MIMAT0004766	hsa-miR-329-3p	MIMAT0001629	hsa-miR-4788	MIMAT0019958	hsa-miR-6785-3p	MIMAT0027471		
hsa-miR-146b-5p	MIMAT0002809	hsa-miR-329-5p	MIMAT0026555	hsa-miR-4791	MIMAT0019963	hsa-miR-6786-3p	MIMAT0027473		
hsa-miR-1470	MIMAT0007348	hsa-miR-330-3p	MIMAT0000751	hsa-miR-4793-3p	MIMAT0019966	hsa-miR-6787-3p	MIMAT0027475		
hsa-miR-1471	MIMAT0007349	hsa-miR-330-5p	MIMAT0004693	hsa-miR-4797-3p	MIMAT0019973	hsa-miR-6788-3p	MIMAT0027477		
hsa-miR-147b	MIMAT0004928	hsa-miR-331-3p	MIMAT0000760	hsa-miR-4798-3p	MIMAT0019975	hsa-miR-6789-5p	MIMAT0027478		
hsa-miR-148a-3p	MIMAT0000243	hsa-miR-331-5p	MIMAT0004700	hsa-miR-4798-5p	MIMAT0019974	hsa-miR-6790-5p	MIMAT0027480		
hsa-miR-148a-5p	MIMAT0004549	hsa-miR-335-3p	MIMAT0004703	hsa-miR-4800-3p	MIMAT0019979	hsa-miR-6791-5p	MIMAT0027482		
hsa-miR-148b	MIMAT0000759	hsa-miR-	MIMAT0000765	hsa-miR-4804-	MIMAT0019985	hsa-miR-6792-	MIMAT0027484		

-3p		335-5p		3p		5p			
hsa-miR-148b-5p	MIMAT0004699	hsa-miR-337-3p	MIMAT0000754	hsa-miR-483-3p	MIMAT0002173	hsa-miR-6793-5p	MIMAT0027486		
hsa-miR-149-5p	MIMAT0000450	hsa-miR-337-5p	MIMAT0004695	hsa-miR-483-5p	MIMAT0004761	hsa-miR-6796-5p	MIMAT0027492		
hsa-miR-150-5p	MIMAT0000451	hsa-miR-338-3p	MIMAT0000763	hsa-miR-484	MIMAT0002174	hsa-miR-6797-3p	MIMAT0027495		
hsa-miR-151a-3p	MIMAT0000757	hsa-miR-339-3p	MIMAT0004702	hsa-miR-485-3p	MIMAT0002176	hsa-miR-6797-5p	MIMAT0027494		
hsa-miR-151a-5p	MIMAT0004697	hsa-miR-339-5p	MIMAT0000764	hsa-miR-485-5p	MIMAT0002175	hsa-miR-6802-3p	MIMAT0027505		
hsa-miR-151b	MIMAT0010214	hsa-miR-33a-3p	MIMAT0004506	hsa-miR-486-5p	MIMAT0002177	hsa-miR-6802-5p	MIMAT0027504		
hsa-miR-152-3p	MIMAT0000438	hsa-miR-33a-5p	MIMAT0000091	hsa-miR-487a-3p	MIMAT0002178	hsa-miR-6803-3p	MIMAT0027507		
hsa-miR-152-5p	MIMAT0026479	hsa-miR-33b-5p	MIMAT0003301	hsa-miR-487a-5p	MIMAT0026559	hsa-miR-6807-5p	MIMAT0027514		
hsa-miR-	MIMAT00	hsa-miR	MIMAT0000750	hsa-miR-	MIMAT000	hsa-miR-	MIMAT0027526		

153-3p	00439	-340-3p		487b-3p	3180	6813-5p			
hsa-miR-1537-3p	MIMAT0007399	hsa-miR-340-5p	MIMAT0004692	hsa-miR-487b-5p	MIMAT0026614	hsa-miR-6815-5p	MIMAT0027530		
hsa-miR-154-3p	MIMAT0000453	hsa-miR-342-3p	MIMAT0000753	hsa-miR-489-3p	MIMAT0002805	hsa-miR-6819-5p	MIMAT0027538		
hsa-miR-154-5p	MIMAT0000452	hsa-miR-345-5p	MIMAT0000772	hsa-miR-490-3p	MIMAT0002806	hsa-miR-6820-3p	MIMAT0027541		
hsa-miR-155-3p	MIMAT0004658	hsa-miR-34a-3p	MIMAT0004557	hsa-miR-490-5p	MIMAT0004764	hsa-miR-6821-5p	MIMAT0027542		
hsa-miR-155-5p	MIMAT0000646	hsa-miR-34a-5p	MIMAT0000255	hsa-miR-491-3p	MIMAT0004765	hsa-miR-6823-3p	MIMAT0027547		
hsa-miR-15a-3p	MIMAT0004488	hsa-miR-34c-5p	MIMAT0000686	hsa-miR-491-5p	MIMAT0002807	hsa-miR-6823-5p	MIMAT0027546		
hsa-miR-15a-5p	MIMAT0000068	hsa-miR-3529-3p	MIMAT0022741	hsa-miR-493-3p	MIMAT0003161	hsa-miR-6825-5p	MIMAT0027550		
hsa-miR-15b-3p	MIMAT0004586	hsa-miR-3591-5p	MIMAT0019876	hsa-miR-493-5p	MIMAT0002813	hsa-miR-6826-5p	MIMAT0027552		
hsa-	MIM	hsa-	MIMAT	hsa-	MIM	hsa-	MIMAT		

miR-15b-5p	AT0000417	miR-3605-3p	0017982	miR-494-3p	AT0002816	miR-6829-5p	0027558		
hsa-miR-16-1-3p	MIMAT0004489	hsa-miR-3605-5p	MIMAT0017981	hsa-miR-494-5p	MIMAT0026607	hsa-miR-6830-5p	MIMAT0027560		
hsa-miR-16-2-3p	MIMAT0004518	hsa-miR-3607-3p	MIMAT0017985	hsa-miR-495-3p	MIMAT0002817	hsa-miR-6831-5p	MIMAT0027562		
hsa-miR-16-5p	MIMAT0000069	hsa-miR-3609	MIMAT0017986	hsa-miR-495-5p	MIMAT0022924	hsa-miR-6832-5p	MIMAT0027564		
hsa-miR-17-3p	MIMAT0000071	hsa-miR-361-3p	MIMAT0004682	hsa-miR-496	MIMAT0002818	hsa-miR-6834-5p	MIMAT0027568		
hsa-miR-17-5p	MIMAT0000070	hsa-miR-361-5p	MIMAT0000703	hsa-miR-497-5p	MIMAT0002820	hsa-miR-6835-5p	MIMAT0027570		
hsa-miR-181a-2-3p	MIMAT0004558	hsa-miR-3610	MIMAT0017987	hsa-miR-498	MIMAT0002824	hsa-miR-6837-3p	MIMAT0027577		
hsa-miR-181a-3p	MIMAT0000270	hsa-miR-3614-3p	MIMAT0017993	hsa-miR-4999-5p	MIMAT0021017	hsa-miR-6840-3p	MIMAT0027583		
hsa-miR-181a-5p	MIMAT0000256	hsa-miR-3614-5p	MIMAT0017992	hsa-miR-499a-5p	MIMAT0002870	hsa-miR-6842-3p	MIMAT0027587		

hsa-miR-181b-2-3p	MIMAT0031893	hsa-miR-3615	MIMAT0017994	hsa-miR-5000-3p	MIMAT0021020	hsa-miR-6844	MIMAT0027589		
hsa-miR-181b-3p	MIMAT0022692	hsa-miR-3616-3p	MIMAT0017996	hsa-miR-5001-3p	MIMAT0021022	hsa-miR-6847-5p	MIMAT0027594		
hsa-miR-181b-5p	MIMAT0000257	hsa-miR-3617-5p	MIMAT0017997	hsa-miR-5001-5p	MIMAT0021021	hsa-miR-6848-5p	MIMAT0027596		
hsa-miR-181c-3p	MIMAT0004559	hsa-miR-3619-3p	MIMAT0019219	hsa-miR-5003-3p	MIMAT0021026	hsa-miR-6849-5p	MIMAT0027598		
hsa-miR-181c-5p	MIMAT0000258	hsa-miR-362-3p	MIMAT0004683	hsa-miR-5003-5p	MIMAT0021025	hsa-miR-6850-5p	MIMAT0027600		
hsa-miR-181d-5p	MIMAT0002821	hsa-miR-362-5p	MIMAT0000705	hsa-miR-5009-5p	MIMAT0021041	hsa-miR-6852-5p	MIMAT0027604		
hsa-miR-182-3p	MIMAT0000260	hsa-miR-3620-5p	MIMAT0022967	hsa-miR-500a-3p	MIMAT0002871	hsa-miR-6854-5p	MIMAT0027608		
hsa-miR-182-5p	MIMAT0000259	hsa-miR-363-3p	MIMAT0000707	hsa-miR-500a-5p	MIMAT0004773	hsa-miR-6855-5p	MIMAT0027610		
hsa-miR-1825	MIMAT0006765	hsa-miR-364	MIMAT0018068	hsa-miR-501-3p	MIMAT0004774	hsa-miR-6856-5p	MIMAT0027612		

		8							
hsa-miR-183-3p	MIMAT0004560	hsa-miR-3651	MIMAT0018071	hsa-miR-501-5p	MIMAT0002872	hsa-miR-6857-3p	MIMAT0027615		
hsa-miR-183-5p	MIMAT0000261	hsa-miR-3652	MIMAT0018072	hsa-miR-5010-3p	MIMAT0021044	hsa-miR-6859-5p	MIMAT0027618		
hsa-miR-184	MIMAT0000454	hsa-miR-3653-3p	MIMAT0018073	hsa-miR-5010-5p	MIMAT0021043	hsa-miR-6862-3p	MIMAT0027626		
hsa-miR-185-3p	MIMAT0004611	hsa-miR-3653-5p	MIMAT0032110	hsa-miR-502-3p	MIMAT0004775	hsa-miR-6865-5p	MIMAT0027630		
hsa-miR-185-5p	MIMAT0000455	hsa-miR-3654	MIMAT0018074	hsa-miR-503-3p	MIMAT0022925	hsa-miR-6866-5p	MIMAT0027632		
hsa-miR-186-3p	MIMAT0004612	hsa-miR-3656	MIMAT0018076	hsa-miR-503-5p	MIMAT0002874	hsa-miR-6869-5p	MIMAT0027638		
hsa-miR-186-5p	MIMAT0000456	hsa-miR-365a-3p	MIMAT0000710	hsa-miR-505-3p	MIMAT0002876	hsa-miR-6871-5p	MIMAT0027642		
hsa-miR-188-5p	MIMAT0000457	hsa-miR-365a-5p	MIMAT0009199	hsa-miR-505-5p	MIMAT0004776	hsa-miR-6873-3p	MIMAT0027647		
hsa-miR-18a-	MIMAT0002891	hsa-miR-	MIMAT0022834	hsa-miR-5088-	MIMAT0021080	hsa-miR-6875-	MIMAT0027651		

3p		365b-3p		5p		3p			
hsa-miR-18a-5p	MIMAT0000072	hsa-miR-3661	MIMAT0018082	hsa-miR-5089-3p	MIMAT0022984	hsa-miR-6876-5p	MIMAT0027652		
hsa-miR-1908-3p	MIMAT0026916	hsa-miR-3662	MIMAT0018083	hsa-miR-5089-5p	MIMAT0021081	hsa-miR-6879-5p	MIMAT0027658		
hsa-miR-1908-5p	MIMAT0007881	hsa-miR-3663-5p	MIMAT0018084	hsa-miR-5095	MIMAT0020600	hsa-miR-6880-3p	MIMAT0027661		
hsa-miR-1909-3p	MIMAT0007883	hsa-miR-3677-3p	MIMAT0018101	hsa-miR-5096	MIMAT0020603	hsa-miR-6880-5p	MIMAT0027660		
hsa-miR-190a-3p	MIMAT0026482	hsa-miR-3679-3p	MIMAT0018105	hsa-miR-516a-5p	MIMAT0004770	hsa-miR-6884-3p	MIMAT0027669		
hsa-miR-190a-5p	MIMAT0000458	hsa-miR-3679-5p	MIMAT0018104	hsa-miR-5187-5p	MIMAT0021117	hsa-miR-6884-5p	MIMAT0027668		
hsa-miR-190b	MIMAT0004929	hsa-miR-3680-5p	MIMAT0018106	hsa-miR-5193	MIMAT0021124	hsa-miR-6886-3p	MIMAT0027673		
hsa-miR-191-3p	MIMAT0001618	hsa-miR-3681-5p	MIMAT0018108	hsa-miR-5196-3p	MIMAT0021129	hsa-miR-6886-5p	MIMAT0027672		
hsa-miR-	MIMAT00	hsa-miR	MIMAT0018110	hsa-miR-	MIMAT000	hsa-miR-	MIMAT0027680		

191-5p	00440	-3682-3p		524-3p	2850	6890-5p			
hsa-miR-1910-5p	MIMAT0007884	hsa-miR-3682-5p	MIMAT0019222	hsa-miR-532-3p	MIMAT0004780	hsa-miR-6891-3p	MIMAT0027683		
hsa-miR-1914-5p	MIMAT0007889	hsa-miR-3684	MIMAT0018112	hsa-miR-532-5p	MIMAT0002888	hsa-miR-6891-5p	MIMAT0027682		
hsa-miR-1915-3p	MIMAT0007892	hsa-miR-3687	MIMAT0018115	hsa-miR-539-3p	MIMAT0022705	hsa-miR-6892-5p	MIMAT0027684		
hsa-miR-192-3p	MIMAT0004543	hsa-miR-3688-3p	MIMAT0018116	hsa-miR-539-5p	MIMAT0003163	hsa-miR-6894-5p	MIMAT0027688		
hsa-miR-192-5p	MIMAT0000222	hsa-miR-369-3p	MIMAT0000721	hsa-miR-541-3p	MIMAT0004920	hsa-miR-6895-3p	MIMAT0027691		
hsa-miR-193a-3p	MIMAT0000459	hsa-miR-369-5p	MIMAT0001621	hsa-miR-541-5p	MIMAT0004919	hsa-miR-7-1-3p	MIMAT0004553		
hsa-miR-193a-5p	MIMAT0004614	hsa-miR-3691-3p	MIMAT0019224	hsa-miR-542-3p	MIMAT0003389	hsa-miR-7-5p	MIMAT0000252		
hsa-miR-193b-3p	MIMAT0002819	hsa-miR-3691-5p	MIMAT0018120	hsa-miR-542-5p	MIMAT0003340	hsa-miR-7107-5p	MIMAT0028111		
hsa-	MIM	hsa-	MIMAT	hsa-	MIM	hsa-	MIMAT		

miR-193b-5p	AT0004767	miR-3692-5p	0018121	miR-543	AT0004954	miR-7108-5p	0028113		
hsa-miR-194-3p	MIMAT0004671	hsa-miR-370-3p	MIMAT0000722	hsa-miR-544a	MIMAT0003164	hsa-miR-7110-3p	MIMAT0028118		
hsa-miR-194-5p	MIMAT0000460	hsa-miR-370-5p	MIMAT0026483	hsa-miR-545-3p	MIMAT0003165	hsa-miR-7112-3p	MIMAT0028122		
hsa-miR-195-3p	MIMAT0004615	hsa-miR-374a-3p	MIMAT0004688	hsa-miR-545-5p	MIMAT0004785	hsa-miR-7113-5p	MIMAT0028123		
hsa-miR-195-5p	MIMAT0000461	hsa-miR-374a-5p	MIMAT0000727	hsa-miR-548a-3p	MIMAT0003251	hsa-miR-7114-5p	MIMAT0028125		
hsa-miR-196a-3p	MIMAT0004562	hsa-miR-374b-3p	MIMAT0004956	hsa-miR-548a-5p	MIMAT0004803	hsa-miR-7154-3p	MIMAT0028219		
hsa-miR-196a-5p	MIMAT0000226	hsa-miR-374b-5p	MIMAT0004955	hsa-miR-548aa	MIMAT0018447	hsa-miR-7158-5p	MIMAT0028226		
hsa-miR-196b-5p	MIMAT0001080	hsa-miR-376a-2-5p	MIMAT0022928	hsa-miR-548ac	MIMAT0018938	hsa-miR-744-3p	MIMAT0004946		
hsa-miR-197-3p	MIMAT0000227	hsa-miR-376a	MIMAT0000729	hsa-miR-548ad-5p	MIMAT0032114	hsa-miR-744-5p	MIMAT0004945		

		-3p							
hsa-miR-197-5p	MIMAT0022691	hsa-miR-376a-5p	MIMAT0003386	hsa-miR-548ae-5p	MIMAT0032115	hsa-miR-758-3p	MIMAT0003879		
hsa-miR-1972	MIMAT0009447	hsa-miR-376b-3p	MIMAT0002172	hsa-miR-548am-5p	MIMAT0022740	hsa-miR-758-5p	MIMAT0022929		
hsa-miR-1973	MIMAT0009448	hsa-miR-376b-5p	MIMAT0022923	hsa-miR-548ap-5p	MIMAT0021037	hsa-miR-760	MIMAT0004957		
hsa-miR-1976	MIMAT0009451	hsa-miR-376c-3p	MIMAT0000720	hsa-miR-548aq-3p	MIMAT0022264	hsa-miR-7641	MIMAT0029782		
hsa-miR-198	MIMAT0000228	hsa-miR-376c-5p	MIMAT0022861	hsa-miR-548aq-5p	MIMAT0022263	hsa-miR-765	MIMAT0003945		
hsa-miR-199a-3p	MIMAT0000232	hsa-miR-377-3p	MIMAT0000730	hsa-miR-548at-5p	MIMAT0022277	hsa-miR-766-3p	MIMAT0003888		
hsa-miR-199a-5p	MIMAT0000231	hsa-miR-377-5p	MIMAT0004689	hsa-miR-548ax	MIMAT0022474	hsa-miR-766-5p	MIMAT0022714		
hsa-miR-199b-3p	MIMAT0004563	hsa-miR-378a-3p	MIMAT0000732	hsa-miR-548ay-3p	MIMAT0025453	hsa-miR-767-5p	MIMAT0003882		
hsa-miR-199b	MIMAT0000263	hsa-miR-	MIMAT0000731	hsa-miR-548ay	MIMAT0025452	hsa-miR-769-	MIMAT0003887		

-5p		378a-5p		-5p		3p			
hsa-miR-19a-3p	MIMAT0000073	hsa-miR-378c	MIMAT0016847	hsa-miR-548b-3p	MIMAT0003254	hsa-miR-769-5p	MIMAT0003886		
hsa-miR-19b-1-5p	MIMAT0004491	hsa-miR-378d	MIMAT0018926	hsa-miR-548c-5p	MIMAT0004806	hsa-miR-770-5p	MIMAT0003948		
hsa-miR-19b-3p	MIMAT0000074	hsa-miR-378e	MIMAT0018927	hsa-miR-548d-3p	MIMAT0003323	hsa-miR-7704	MIMAT0030019		
hsa-miR-200a-3p	MIMAT0000682	hsa-miR-378f	MIMAT0018932	hsa-miR-548d-5p	MIMAT0004812	hsa-miR-7705	MIMAT0030020		
hsa-miR-200a-5p	MIMAT0001620	hsa-miR-378i	MIMAT0019074	hsa-miR-548e-3p	MIMAT0005874	hsa-miR-7706	MIMAT0030021		
hsa-miR-200b-3p	MIMAT0000318	hsa-miR-379-3p	MIMAT0004690	hsa-miR-548e-5p	MIMAT0026736	hsa-miR-7845-5p	MIMAT0030420		
hsa-miR-200b-5p	MIMAT0004571	hsa-miR-379-5p	MIMAT0000733	hsa-miR-548f-5p	MIMAT0026739	hsa-miR-7847-3p	MIMAT0030422		
hsa-miR-200c-3p	MIMAT0000617	hsa-miR-380-3p	MIMAT0000735	hsa-miR-548h-3p	MIMAT0022723	hsa-miR-7854-3p	MIMAT0030429		
hsa-miR-202-5p	MIMAT0002810	hsa-miR-381-3p	MIMAT0000736	hsa-miR-548h-5p	MIMAT0005928	hsa-miR-7976	MIMAT0031179		
hsa-	MIM	hsa-	MIMAT	hsa-	MIM	hsa-	MIMAT		

miR-203a-3p	AT0000264	miR-381-5p	0022862	miR-548i	AT0005935	miR-8485	0033692		
hsa-miR-205-5p	MIMAT0000266	hsa-miR-382-3p	MIMAT0022697	hsa-miR-548j-3p	MIMAT0026737	hsa-miR-874-3p	MIMAT0004911		
hsa-miR-20a-3p	MIMAT0004493	hsa-miR-382-5p	MIMAT0000737	hsa-miR-548j-5p	MIMAT0005875	hsa-miR-874-5p	MIMAT0026718		
hsa-miR-20a-5p	MIMAT0000075	hsa-miR-3909	MIMAT0018183	hsa-miR-548k	MIMAT0005882	hsa-miR-877-3p	MIMAT0004950		
hsa-miR-20b-5p	MIMAT0001413	hsa-miR-3911	MIMAT0018185	hsa-miR-548l	MIMAT0005889	hsa-miR-877-5p	MIMAT0004949		
hsa-miR-21-3p	MIMAT0004494	hsa-miR-3912-3p	MIMAT0018186	hsa-miR-548m	MIMAT0005917	hsa-miR-885-5p	MIMAT0004947		
hsa-miR-21-5p	MIMAT0000076	hsa-miR-3913-3p	MIMAT0019225	hsa-miR-548n	MIMAT0005916	hsa-miR-887-3p	MIMAT0004951		
hsa-miR-210-3p	MIMAT0000267	hsa-miR-3916	MIMAT0018190	hsa-miR-548o-3p	MIMAT0005919	hsa-miR-888-5p	MIMAT0004916		
hsa-miR-210-5p	MIMAT0026475	hsa-miR-3925-5p	MIMAT0018200	hsa-miR-548o-5p	MIMAT0022738	hsa-miR-889-3p	MIMAT0004921		

hsa-miR-2110	MIMAT0010133	hsa-miR-3928-3p	MIMAT0018205	hsa-miR-548t-3p	MIMAT0022730	hsa-miR-889-5p	MIMAT0026719		
hsa-miR-2114-5p	MIMAT0011156	hsa-miR-3929	MIMAT0018206	hsa-miR-548t-5p	MIMAT0015009	hsa-miR-9-3p	MIMAT0000442		
hsa-miR-2116-5p	MIMAT0011160	hsa-miR-3934-3p	MIMAT0022975	hsa-miR-548u	MIMAT0015013	hsa-miR-9-5p	MIMAT0000441		
hsa-miR-212-3p	MIMAT0000269	hsa-miR-3934-5p	MIMAT0018349	hsa-miR-548w	MIMAT0015060	hsa-miR-92a-1-5p	MIMAT0004507		
hsa-miR-212-5p	MIMAT0022695	hsa-miR-3935	MIMAT0018350	hsa-miR-548z	MIMAT0018446	hsa-miR-92a-3p	MIMAT0000092		
hsa-miR-215-5p	MIMAT0000272	hsa-miR-3939	MIMAT0018355	hsa-miR-549a	MIMAT0003333	hsa-miR-92b-3p	MIMAT0003218		
hsa-miR-216a-3p	MIMAT0022844	hsa-miR-3940-3p	MIMAT0018356	hsa-miR-550a-3p	MIMAT0003257	hsa-miR-92b-5p	MIMAT0004792		
hsa-miR-216a-5p	MIMAT0000273	hsa-miR-3940-5p	MIMAT0019229	hsa-miR-551a	MIMAT0003214	hsa-miR-93-3p	MIMAT0004509		
hsa-miR-217	MIMAT0000274	hsa-miR-394	MIMAT0018358	hsa-miR-556-3p	MIMAT0004793	hsa-miR-93-5p	MIMAT0000093		

		2-5p							
hsa-miR-218-5p	MIMAT0000275	hsa-miR-3944-3p	MIMAT0018360	hsa-miR-5581-3p	MIMAT0022276	hsa-miR-933	MIMAT0004976		
hsa-miR-219a-1-3p	MIMAT0004567	hsa-miR-3960	MIMAT0019337	hsa-miR-5581-5p	MIMAT0022275	hsa-miR-935	MIMAT0004978		
hsa-miR-219b-5p	MIMAT0019747	hsa-miR-409-3p	MIMAT0001639	hsa-miR-5583-3p	MIMAT0022282	hsa-miR-937-3p	MIMAT0004980		
hsa-miR-22-3p	MIMAT0000077	hsa-miR-409-5p	MIMAT0001638	hsa-miR-5584-3p	MIMAT0022284	hsa-miR-937-5p	MIMAT0022938		
hsa-miR-22-5p	MIMAT0004495	hsa-miR-410-3p	MIMAT0002171	hsa-miR-5584-5p	MIMAT0022283	hsa-miR-941	MIMAT0004984		
hsa-miR-221-3p	MIMAT0000278	hsa-miR-410-5p	MIMAT0026558	hsa-miR-5585-3p	MIMAT0022286	hsa-miR-942-3p	MIMAT0026734		
hsa-miR-221-5p	MIMAT0004568	hsa-miR-411-3p	MIMAT0004813	hsa-miR-5585-5p	MIMAT0022285	hsa-miR-942-5p	MIMAT0004985		

Table S2 the predicted targets of miR-212/132 using bioinformatics algorithms

**Gene name**	**Gene name**	**Gene name**	**Gene name**	**Gene name**	**Gene name**

FBXO42	RDX	SPRED1	TRIB2	FBXW7	SHH
HNRNPR	ARHGAP32	THBS1	SPAST	PCGF3	TMEM106B
SRSF10	TEAD1	RTF1	SLC30A6	TADA2B	CREB5
SNIP1	GTF2H1	MAPKBP1	GEMIN6	STIM2	CASD1
MTF1	CAPRIN1	CTDSPL2	MEIS1	SLAIN2	BRI3
CC2D1B	FKBP2	FGF7	KCMF1	DCUN1D4	MEPCE
ZCCHC11	KDM5A	USP8	TCF7L1	PDGFRA	CBLL1
DAB1	SOX5	TLN2	GPD2	ENPEP	FOXP2
ZNF644	H3F3C	ARIH1	SCN2A	SPRY1	MDFIC
TMED5	ARF3	ANKRD34C	CSRNP3	PCDH10	CALU
EXTL2	ZFC3H1	MEF2A	LZTS3	USP38	TMEM178B
CSDE1	OSBPL8	DCUN1D3	RNF24	GAB1	FAM167A
SV2A	KITLG	ARHGAP17	SLC23A2	HAPLN1	ZNF395
MEX3A	POC1B	MAPK3	RBM12	EDIL3	PPP2CB
ARHGEF11	POC1B-GALNT4	C16orf87	FITM2	CHD1	KAT6A
DCAF8	FBXO21	DNAJA2	TAF4	NREP	PDE7A
SCYL3	PXN	SALL1	CBFA2T2	FEM1C	MMP16
KIF21B	SPPL3	DYNC1LI2	STX16	IRF1	AZIN1
KDM5B	AEBP2	NFAT5	ADAMTS5	AFF4	SAMD12
ANGEL2	ETNK1	HSBP1	BRWD1	HBEGF	HAS2
CDC42BPA	ARID2	ABR	MAPK1	DIAPH1	AGO2
ARID4B	DAZAP2	CRK	PRR14L	DPYSL3	TACC1
AKT3	USP15	NCOR1	DGCR8	GHR	LYN
HNRNPU	SRGAP1	SREBF1	HIC2	ISL1	SGK3
OTUD3	LEMD3	FBXL20	CCDC117	SNX18	ZFHX4
HMGN2	HMGA2	BRCA1	HMGXB4	ZSWIM6	E2F5
ARID1A	DYRK2	ZNF652	EP300	RASA1	SDC2
ZDHHC18	CPSF6	ANKRD40	SRGAP3	PAM	FAM91A1
AGO1	CNOT2	SRSF1	IQSEC1	PRDM6	PHF20L1
AKIRIN1	SOCS2	PSMD12	CELSR3	SEC24A	RFX3
PRKAA2	HIP1R	H3F3B	PRICKLE2	SMAD5	NFIB
NFIA	FOXO1	KCNJ12	FOXP1	EGR1	BNC2
PTBP2	SPRYD7	NLK	ZBTB20	PAIP2	TMEM2
GABPB2	DACH1	ZNF207	GSK3B	SAP30L	GOLM1
PEA15	MYCBP2	MAPT	KPNA1	LSM11	PTCH1
CAMSAP2	PAN3	KAT7	RAB6B	ATXN1	POLE3
BTG2	RB1	DYNLL2	SERP1	CDK19	NACC2
SRGAP2	TFDP1	MAP3K3	MECOM	FRK	SEC16A

HSD11B1	CHD8	ROCK1	SETD5	CTGF	RORB
KCNK2	NOVA1	ANKRD29	SLC6A1	CITED2	TMEFF1
MIA3	PRKD1	ZNF521	TMEM43	SOX4	MSANTD3 -TMEFF1
FBXO28	HECTD1	SS18	ITGA9	CDKN1A	KIAA1958
ZBTB18	SPTSSA	B4GALT6	ACVR2B	NFYA	SLC31A1
DESI2	CFL2	SMAD2	ABHD5	TJAP1	GAPVD1
SEPHS1	FOXA1	ZNF516	RAD54L2	TTK	ZBTB34
NMT2	CNIH1	VAPA	FLNB	CYB5R4	FUBP3
EPC1	GMFB	TAF4B	RPP14	ZNF292	OLFM1
SLC25A28	TIMM9	UQCRFS1	C3orf14	LIN28B	ARX
EBF3	SIX4	C19orf47	ATXN7	FOXO3	AMOT
NET1	PPP2R5E	CRTC1	MITF	AMD1	MMGT1
CELF2	MAP3K9	ADCY3	BBX	PDE7B	MECP2
SIRT1	ELMSAN1	DNMT3A	RAP2B	WTAP	USP9X
BMPR1A	FOXN3	ASXL2	SEC62	QKI	DUSP9
PTEN	BTBD7	PPM1G	PIK3CA	GNA12	SHH
BTAF1	CLMN	SOS1	FXR1	ETV1	TMEM106 B
SEMA4G	PNN	CCDC88A	EIF4A2	SKAP2	CREB5
VTI1A	DAAM1	RAB1A	LMLN	PSMA2	CASD1
TCF7L2	PPP2R5C	GTDC1	SLBP	NUDCD3	BRI3
DKK3	MTMR10	ZEB2	CLOCK	POM121C	MEPCE
SOX6	SLC12A6	RND3	GRSF1	YWHAG	CBLL1
ARFGAP2	MEIS2	NR4A2	PYURF	GIGYF1	FOXP2
ATL3	SLTM	ACVR1	H2AFZ	CCDC71L	MDFIC
SHANK2	BNIP2	SATB2	PPP3CA	NRCAM	CALU

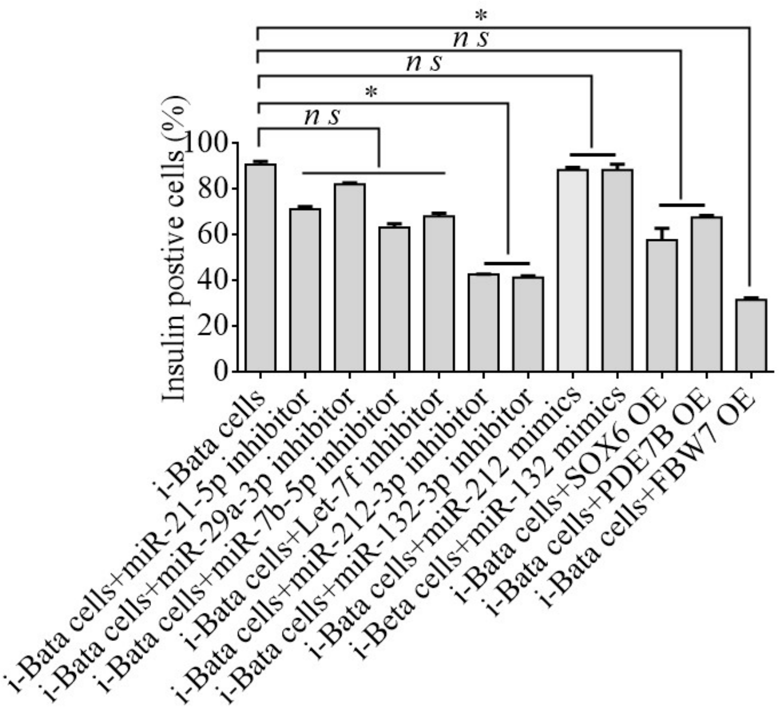

Figure S1 Statistical analysis of the rate of insulin positive cell under different treatment.

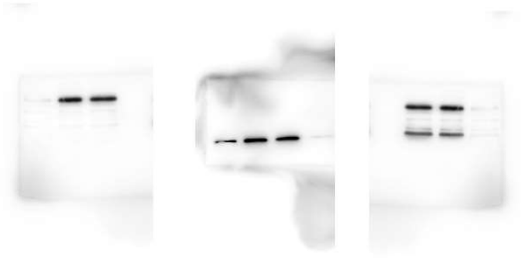

Figure 1-WB whole image

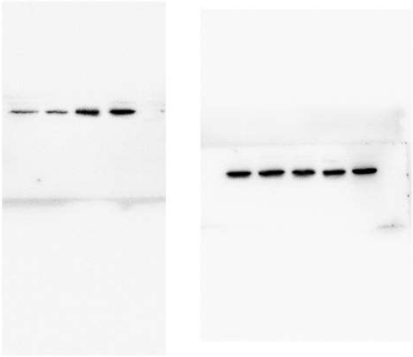

Figure 6-WB whole image

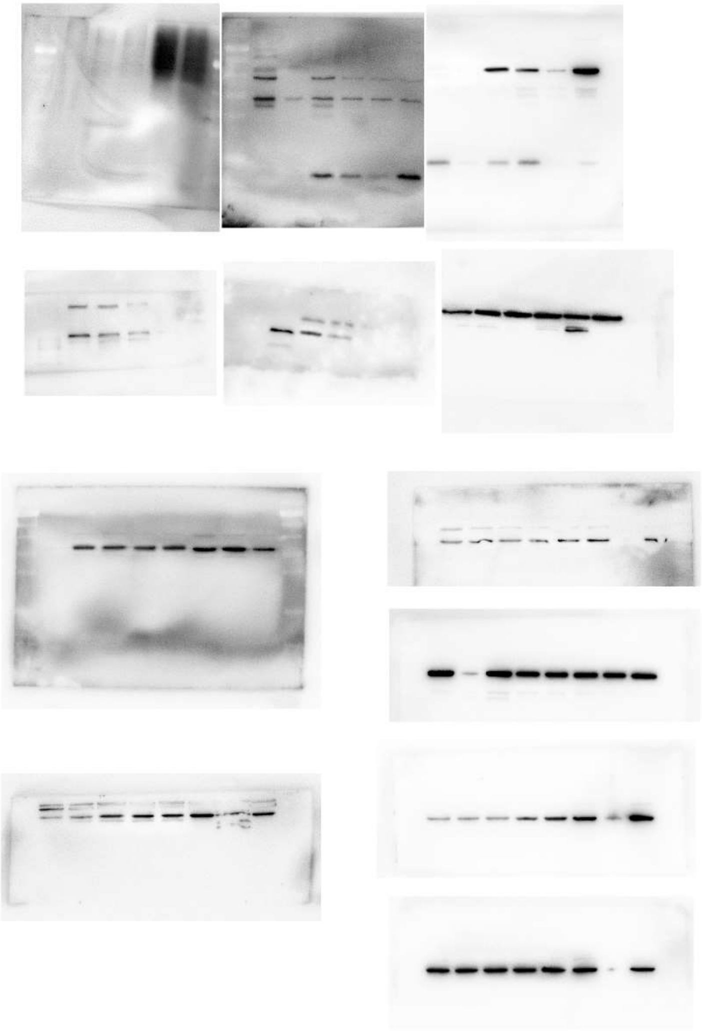

Figure 7-WB whole image

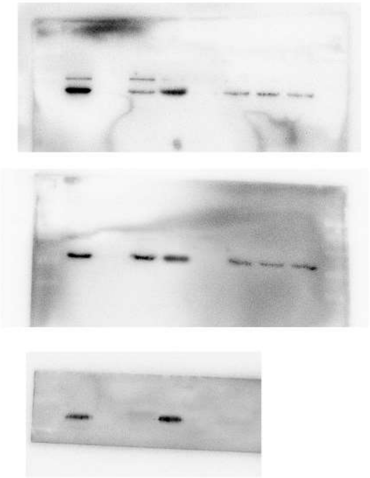

Figure 8-WB whole image
